# Nanopore sequencing reveals coordinated host and microbiome responses across barley genotypes

**DOI:** 10.1186/s12915-026-02688-3

**Published:** 2026-07-29

**Authors:** Bennet Rohan Fernando Devasahayam, Thomas McNeil, Tesfaye Wubet, Thomas Schmutzer

**Affiliations:** 1 Breeding Informatics, Chair of Plant Breeding, Faculty of Natural Sciences III, Martin Luther University Halle-Wittenberg, Karl-Freiherr-von-Fritsch-Str. 4, Halle (Saale), 06120 Germany; 2 Department of Community Ecology, Helmholtz Centre for Environmental Research—UFZ, Theodor-Lieser-Str. 4, Halle (Saale), 06120 Germany; 3 German Centre for Integrative Biodiversity Research (iDiv) Halle-Jena-Leipzig, Puschstrasse 4, Leipzig, 04103 Germany; 4 Institute of Biology/Geobotany and Botanical Garden, Martin Luther University Halle-Wittenberg, Am Kirchtor 1, Halle (Saale), 06108 Germany

**Keywords:** Rhizosphere microbiome, Long-read nanopore sequencing, Metagenomics, Transcriptomics, Host-microbiome interactions

## Abstract

**Background:**

Barley (*Hordeum vulgare* L.) provides a suitable model for studying domestication-driven plant-microbiome interactions. Although wild, landrace, and modern genotypes host distinct rhizosphere communities, the extent to which roots and microbes reciprocally influence each other remains unclear. Here, we applied an integrated multi-omics approach combining long-read metagenomics, root transcriptomics, and plant genomics to understand genotype-specific host-microbiome coordination.

**Results:**

Oxford Nanopore whole metagenome sequencing (WMS) revealed genotype-associated shifts in rhizosphere communities across seasons. Functional profiling showed a conserved metabolic backbone including amino acid metabolism, energy production, and secondary metabolite biosynthesis, alongside genotype-dependent variation in carbohydrate metabolism and transport-associated pathways. Genome-resolved analysis through metagenome-assembled genomes (MAGs) further detailed the taxonomic and functional architecture of key rhizosphere lineages. Root transcriptome profiling identified extensive differential expression associated with microbial perception, signaling, defense, and metabolic processes. Integration of host and microbiome data revealed coordinated molecular patterns, indicating that barley genotypes are associated with distinct microbial assemblages and corresponding transcriptional responses.

**Conclusions:**

These findings indicate that domestication has shaped coordinated associations between barley genotypes and their rhizosphere microbiomes, reflected in both microbial community composition and host transcriptional regulation. This work provides new insights into the evolutionary tuning of plant-microbiome relationships and highlights opportunities for microbiome-informed strategies in barley improvement.

**Supplementary Information:**

The online version contains supplementary material available at 10.1186/s12915-026-02688-3.

## Background

Barley (*Hordeum vulgare* L.) represents one of the earliest domesticated cereal crops, with archeological evidence tracing its cultivation back more than 10,000 years in the Fertile Crescent [1, 2]. Its domestication from the wild progenitor *Hordeum spontaneum* led to the establishment of landraces adapted to diverse agroecological environments, and later to modern cultivars shaped by intensive breeding [3, 4]. In the context of this domestication trajectory, barley genotypes can be broadly categorized into three groups viz. wild accessions representing the ancestral gene pool, landraces reflecting intermediate domestication and local adaptation, and modern elite cultivars shaped by recent breeding. These groups capture a gradient of domestication history and, in aggregate, are expected to differ in genetic diversity and the frequency of domestication-associated alleles, although genetic relatedness within and between groups can vary depending on accession history [3, 5]. Understanding how this domestication gradient is reflected requires genomic resources that allow accurate comparison of gene content, structural variation, and transcriptional responses across genotypes. The Hi-C guided Morex assembly produced the first chromosome-scale reference for barley (~ 5 Gb), providing a robust scaffold for read mapping, variant discovery, and gene localization, and establishing a common coordinate system for downstream analyses [6]. Moving beyond a single reference, the first barley pan-genome, which included 20 varieties spanning wild accessions, landraces, and elite cultivars, revealed extensive presence or absence variation and large inversion polymorphisms in elite germplasm that could not be detected from a single genome [7]. A recent expanded pan-genome with 76 long-read assemblies and short-read data for 1315 genotypes now charts structural variants and copy number–rich loci across domestication space, substantially improving the catalog of allelic diversity for breeding [8]. At gene-space resolution, exome capture platforms and studies on diverse panels including 267 geo-referenced landraces/wilds, ~ 371 domesticated lines, and other regional panels uncovered adaptive variants tied to environment and agronomic performance, providing cost-effective variant discovery for large cohorts [3]. Wild barley de novo assemblies complement these resources by exposing structural and gene-content differences relative to Morex, refining domestication inferences and improving read-mapping for wild introgressions [9]. Contemporary reviews summarize this shift from single-reference to pangenomic thinking and outline how structural variation (SV) drives phenotypic diversification under domestication and improvement [10]. Methodologically, these assemblies and panels translate into concrete bioinformatics practice such as aligning reads to chromosome-scale pseudomolecules, and discovery of structural variants with long-reads/graph genomes, while exome data enable high-density single-nucleotide polymorphism (SNP) matrices for genome-wide association study (GWAS) and genomic prediction [11]. Together, these genomic resources enable precise dissection of transitions from wild to landraces to elite cultivars, including domestication footprints at candidate loci affecting adaptation, quality, and stress response [7]. As barley genomics converges on graph- and pan-reference frameworks, downstream analyses such as variant calling, GWAS and expression quantitative trait locus (eQTL) will increasingly account for non-reference haplotypes which is an important consideration when relating host genotype to rhizosphere microbiome and root transcriptional phenotypes in wild, landrace, and modern genotypes.

Beyond the genetic and agronomic importance of barley, recent studies suggest that domestication-driven selection also extends belowground, influencing how plant roots interact with the rhizosphere microbiome [12, 13]. Genotype-associated differences in rhizosphere microbiome composition are thought to arise through multiple, non-mutually exclusive biological mechanisms. Variation in root exudate quantity and composition can alter nutrient availability and chemoattraction in the rhizosphere, thereby influencing microbial recruitment and activity [14]. In addition, genotype-dependent differences in root architecture, development, and growth dynamics can modify physical microhabitats along the root surface. Hormonal and transporter networks linking nutrition, defense, and stress responses may also indirectly influence microbiome assembly [15]. Importantly, these host-associated effects frequently interact with environmental conditions, and the relative contribution of genotype versus soil and year remains incompletely resolved under field conditions. In cereals such as rice, wheat, and maize, rhizosphere microbial composition has been shown to vary with genotype, developmental stage, and environmental conditions [16–18]. Although barley is a model crop with extensive genetic resources, our understanding of its rhizosphere microbiome lags behind other cereals, with recent studies only beginning to uncover the diversity and host-specificity of its microbial communities [19]. Given the central role of the rhizosphere in mediating plant–microbe interactions, understanding how microbiome composition varies across barley types offers a unique opportunity to link host genetic background, microbial diversity, and plant performance.

Domestication and modern breeding have not only reshaped the genetic architecture of crops but also influenced their interactions with soil microbial communities. However, the magnitude and consistency of domestication-associated microbiome patterns vary across crops and environments, and genotype effects often explain a smaller proportion of community variation than local soil and seasonal conditions. Comparative studies across crops indicate that both wild relatives and domesticated cultivars assemble distinct and functionally diverse rhizosphere microbiomes, with patterns largely determined by host genotype and domestication status [20, 21]. Importantly, wild barley genotypes have been reported to enrich for microbial taxa associated with nutrient mobilization and stress resilience, whereas elite cultivars tend to assemble more streamlined microbial communities [22]. These findings highlight that domestication-driven shifts in microbial diversity and functionality may have contributed to changes in plant adaptability, raising the question of how variation in barley types continues to influence host-microbiome associations under field conditions. In barley, the host genotype accounts for a smaller proportion of rhizosphere variation than the local soil microhabitat, yet it still exerts a consistent influence on microbial community composition [12]. The differences between wild and modern barley genotypes have been linked to specific host loci, most notably the QRMC-3HS locus on chromosome 3H, which influences the recruitment of diverse bacterial taxa and explains up to 20% of their variation. Bacteria preferentially recruited by the wild allele at this locus include *Variovorax*, *Holophaga*, *Sorangium*, *Tahibacter*, and *Rhodanobacter*, representing functionally diverse groups involved in complex carbon turnover [19]. By contrast, elite cultivars often show stronger enrichment of Actinobacteria, a phylum associated with antimicrobial production and stress-adapted lifestyles, consistent with a more filtered microbiome [23, 24]. Metagenome-assembled genomes (MAGs) from barley rhizospheres further reveal that the partitioning of key microbial functions, including nitrogen metabolism and transporter repertoires, is strongly genotype-dependent, indicating that domestication has not only influenced the microbial community structure but also their functional potential [25]. Plant-soil feedback experiments reinforce this view, showing that a phylogenetically diverse microbial consortium supports optimal growth of elite barley under nitrogen limitation, emphasizing that functional diversity remains advantageous even for modern genotypes [22]. Mechanistically, immune-related host factors are likely central in this process. For instance, QRMC-3HS harbors a candidate nucleotide-binding leucine-rich repeat (NLR) gene BaRT2v18chr3HG123500 with structural variation across the barley pan-genome, raising the possibility that immune-related loci may contribute to microbiome recruitment, although direct causal links between immune genes and domestication-driven microbiome shifts remain to be functionally validated [19]. Earlier studies also identified root-enriched families such as *Comamonadaceae*, *Flavobacteriaceae*, and *Rhizobiaceae* and found positive selection on microbial protein families involved in pathogenesis, secretion, phage interactions, and nutrient mobilization which are signatures of long-term host-microbe co-adaptation [12]. Differences among barley cultivars also take place at the strain and functional levels. For example, inoculation with matched rhizosphere extracts or *Pseudomonas* synthetic communities (SynComs) produces cultivar-specific growth responses, suggesting that elite genotypes respond to narrower sets of microbial partners compared to wild or landrace backgrounds [26]. Collectively, cross-crop studies highlight that domestication has reshaped the composition and functional potential of rhizosphere microbiomes, consistently influencing the abundance of taxa comprising key microbial functional guilds [24]. Altogether, these observations support a model where wild barley maintains broader microbial functional repertoires, while elite cultivars exhibit selective filtering that favors a leaner set of partners, with trade-offs for resilience vs input-dependent performance.

Building on the domestication-driven patterns in the rhizosphere microbiome, it is essential to understand how these microbial communities induce transcriptional changes within barley roots. Transcriptome studies on barley roots inoculated with species of bacteria show that colonization can rapidly rewire the barley transcriptome activating defense-associated pathways, transport processes, and signaling modules [27]. Complementary work demonstrates that barley differentiates its transcriptional response to distinct beneficial taxa including *Ensifer*, *Pantoea*, and *Pseudomonas*, indicating that host signaling discriminates among commensals and promotes tailored root programs rather than a generic beneficial microbe response [28]. In the context of symbiosis, arbuscular mycorrhizal fungi (AMF) modulate nutrient-uptake circuits in barley roots. For instance, AMF inoculation increased grain Zn and altered root ZIP transporter expression in a modern barley cultivar, a clear instance where microbial association reshapes host ion-homeostasis genes [29]. In addition, genetic gating of these interactions is evident from the *MLO* (*Mildew Resistance Locus O*), which is classically known for conferring broad-spectrum, stable resistance to powdery mildew in barley [30]. Beyond this canonical function, *MLO* has recently been shown to regulate colonization by arbuscular mycorrhizal fungi and root endophytes [31], indicating that immune-related loci can influence both rhizosphere assembly and host transcriptional responses. Cultivar-specific differences in barley also influence microbiota-host signaling, as seen in the recruitment and activity of *Pseudomonas* populations accompanied by microbial transcriptome shifts, indicating that host genotype determines distinct transcriptional trajectories during colonization [26]. Although most barley transcriptome studies address abiotic stress, they consistently reveal root modules such as hormone, transport, redox, and cell-wall pathways that are also engaged during microbe-plant interactions, with parallel evidence from AMF studies in wheat and meta-analyses linking biotic stress and hormone networks [32, 33]. Recent studies also highlight defense priming and nutrition-linked transcriptional shifts in barley exposed to rhizobacteria, connecting root gene expression to whole-plant outcomes such as reduced pest burdens, emphasizing that microbial effects are routed through host transcriptional control layers [34]. Methodologically, cell-type-resolved and spatial transcriptomics are emerging to dissect which root tissues execute these programs during beneficial colonization, promising sharper resolution of barley’s microbe-responsive gene networks [35]. Together, these findings support a model in which microbial association states including commensal, or mutualist are read out as distinct barley root transcriptional fingerprints spanning immune receptors, secondary signaling, phytohormone circuitry, transporters, and secretory machinery. Importantly, they also align with the domestication signal which indicates that host genotype may influence both rhizosphere community assembly and the associated architecture of transcriptional responses.

In order to address genotype-associated variation in rhizosphere microbiome and host-microbe interactions, we applied a multi-omics framework integrating long-read whole metagenome sequencing (WMS) of the rhizosphere microbiome, root transcriptome profiling and plant genomics across different barley genotypes spanning a domestication gradient. Based on prior reports that domestication alters root traits, immune signaling, and resource allocation, we hypothesized that barley genotypes would recruit rhizosphere microbiomes with domestication gradient-dependent taxonomic and functional signatures. We further hypothesized that these microbiome differences would be reflected in host transcriptional responses, particularly in pathways related to defense, signaling, and transport. By combining multi-omics, this study aims to assess how genotype-associated variation corresponds with coordinated host-microbiome interactions under field conditions, while accounting for seasonal variation.

## Results

### Rhizosphere microbial community assembly

To assess genotype-associated variation in rhizosphere microbiome composition, we initially profiled rhizosphere samples from 21 barley genotypes representing wild accessions, landraces, and modern cultivars grown under field conditions. Bulk soil collected from unplanted areas within the same field served as a reference control to characterize the background soil microbial community. Illumina MiSeq-based short-read profiling of rhizosphere soil microbial communities from 21 barley genotypes (seven genotypes per domestication group) and two controls revealed significant differences in community composition grouped by domestication status (PERMANOVA: R^2^ = 0.2273, *p* < 0.001). Pairwise comparisons further showed that all three barley status groups differed significantly from the controls, with the largest dissimilarity observed for wild (31.5%), followed by landrace (25%) and modern (10.8%) barley genotypes. While modern genotypes differed significantly from both wild and landrace groups, no significant difference was detected between wild and landrace genotypes. Consistent with these results, PCoA ordination demonstrated separation among the genotype groups, with the first two axes explaining 25.5% of the variation in rhizosphere bacterial community composition (Additional file 1: Fig. S1; Additional file 2: Table. S1). Together, these findings indicate that domestication status is a major determinant of rhizosphere microbial community structure across the barley genotypes.

Based on the observed community patterns across domestication groups, a representative subset of five genotypes was selected for in-depth long-read whole metagenome sequencing (WMS). This subset comprised of two wild (HID0144 and HID0380) and two landrace (HID1029 and HID1104) genotypes, and one modern cultivar (Gretchen), reflecting contrasting microbiome profiles observed in the initial short-read analysis. This selection enabled detailed taxonomic and functional characterization of rhizosphere microbiomes across selected barley genotypes using Oxford Nanopore (ONT) sequencing.

### Oxford Nanopore long-read metagenome sequencing of rhizosphere microbiome

ONT sequencing of the selected genotypes generated high-quality long-read data from rhizosphere samples collected across two field seasons (2024 and 2025). Sequencing quality metrics including read length distributions, sequencing depth, and total yield are provided in the supplementary information (Additional file 1: Fig. S2a—d; Additional file 2: Tables. S2 and S3). Taxonomic classification revealed that bacterial species predominated across all samples, whereas fungal reads showed lower classification rates, consistent with limited representation of fungal genomes in current reference databases [36] (Additional file 1: Fig. S2e and f).

### Taxonomic composition of the barley rhizosphere microbiomes indicates genotype-dependent community shifts

**Fig. 1:**
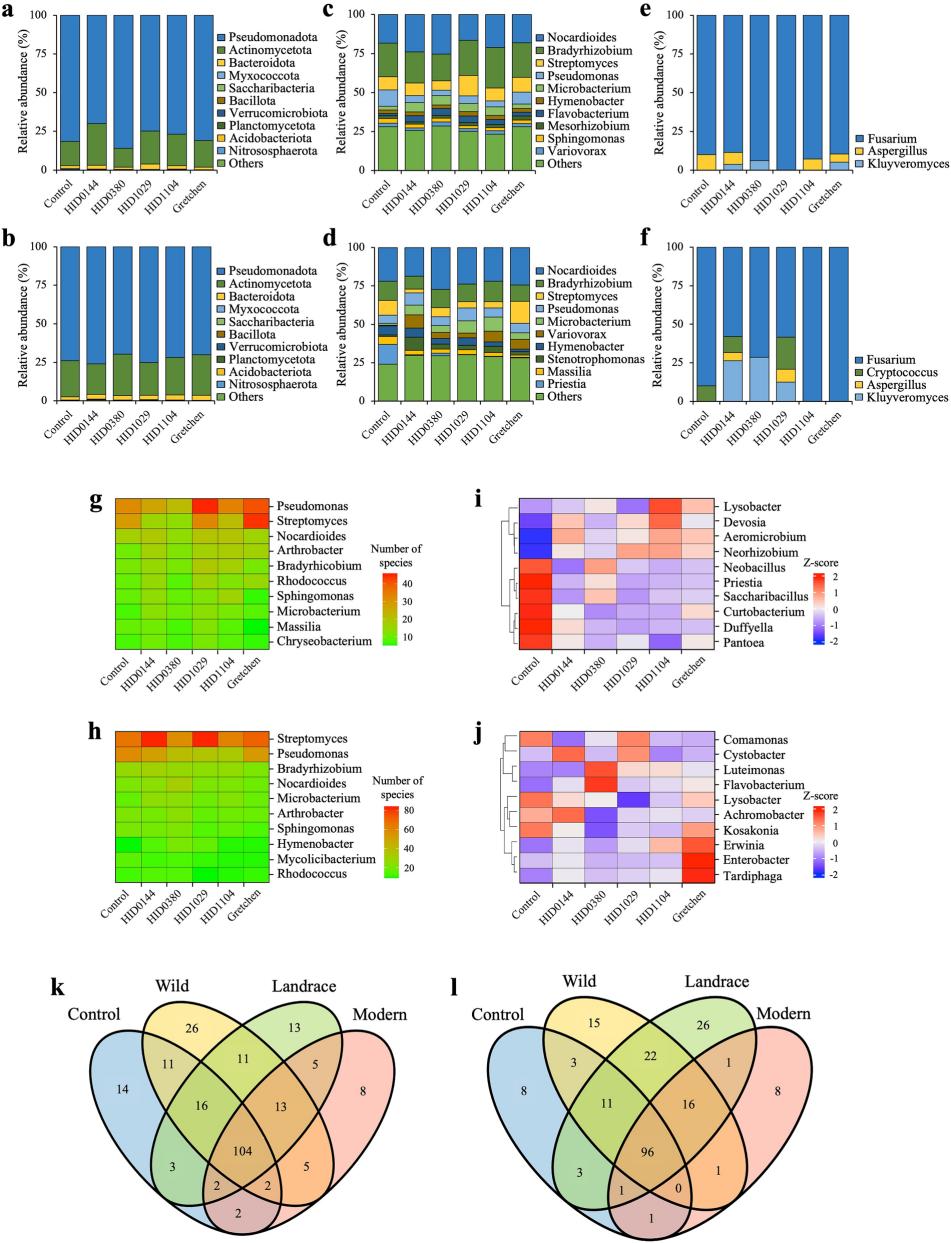
Oxford Nanopore whole-metagenome sequencing (WMS) reveals taxonomic composition of bacterial and fungal communities in rhizosphere microbiomes. **a**,** b** Stacked bar plots showing the relative abundance of the top 10 bacterial phyla identified from whole-metagenome sequencing in 2024 (**a**) and 2025 (**b**), illustrating the dominance of *Pseudomonadota* and *Actinomycetota* across all samples. **c, d** Stacked bar plots depicting the genus-level composition of the top 10 bacterial communities in 2024 (**c**) and 2025 (**d**), highlighting *Nocardioides*, *Bradyrhizobium*, and *Streptomyces* as consistently dominant taxa. **e, f** Stacked bar plots showing the relative abundance of major fungal genera detected by WMS in 2024 (**e**) and 2025 (**f**), with *Fusarium* dominating across genotypes. Relative abundances are shown after excluding unclassified reads and renormalizing classified taxa to 100%. **g, h** Heatmaps illustrating species richness within the top 10 bacterial genera, represented by the number of detected species per genus in 2024 (**g**) and 2025 (**h**), with *Pseudomonas* and *Streptomyces* exhibiting highest species-level diversity. **i, j** Differential abundance heatmaps showing bacterial genera significantly enriched or depleted relative to control soil in 2024 (**i**) and 2025 (**j**). Colors represent ***Z***-scores derived from normalized log₂ fold changes. **k, l** Venn diagrams illustrating shared and unique bacterial genera among control, wild accessions, landraces, and the modern cultivar Gretchen in 2024 (**k**) and 2025 (**l**). Overlapping regions denote the core microbiota common to all groups. Each genotype is represented by two biological replicates

The rhizosphere microbiome plays an important role in plant growth, nutrient turnover, and stress resilience [14, 37]. The composition and structure of root-associated microbial communities are key determinants of plant fitness [38] and have strongly been reported to vary in relation to domestication and breeding. To investigate genotype-specific microbial community structures and their diversity across seasons, rhizosphere samples from wild accessions HID0144 and HID0380, landraces HID1029 and HID1104, and the modern cultivar Gretchen were analyzed at different taxonomic ranks. For visualization purposes, only classified reads were included, and relative abundances were normalized to 100% after excluding unclassified reads (Fig. 1). Taxonomic profiles were generated using Kraken2 with a confidence threshold of 0.1, which was selected to maximize sensitivity for detecting low-abundance taxa in complex soil metagenomes.

At the phylum level, the rhizosphere bacterial communities of different barley genotypes were dominated by Pseudomonadota and Actinomycetota, which together accounted for the majority of reads across both field seasons. These two phyla formed a stable microbiome shared among wild, landrace, and modern barley genotypes. Temporal variation was evident, with Actinomycetota increasing in relative abundance from 12.1% in 2024 to 27% in 2025 (Fig. 1a and b). At the genus level, taxa including *Nocardioides*, *Bradyrhizobium*, and *Streptomyces* were consistently dominant across all barley types. While these genera were consistently present across all samples, their relative abundance varied markedly among genotypes and between years. Interestingly, between years, the relative abundance of *Streptomyces* increased in Gretchen, from 3.5% in 2024 to 14.1% in 2025, suggesting temporal shifts in this actinobacterial genus (Fig. 1c and d). In contrast, *Bradyrhizobium* declined by approximately 10% over the same period, possibly reflecting environmental influences on nitrogen-fixing microbial populations. These trends highlight shifts in rhizosphere bacterial composition influenced by both host genotype and environmental changes between years. Such genotype-dependent patterns are consistent with previous findings linking host genetic background to microbial recruitment [12, 20]. To assess the robustness of these genus-level patterns to taxonomic stringency, we repeated the Kraken2 classification using higher-level confidence thresholds of 0.5 and 0.7 (Additional file 1: Fig. S3). At a threshold of 0.5, pronounced differences in bacterial genus composition were observed between 2024 and 2025 (Fig. S3a and c), with few genera present at 0.1 no longer detected and genotype-specific patterns becoming more distinct. Applying a threshold of 0.7 yielded profiles largely similar to those obtained at 0.5, but with consistent genotype-specific shifts within each year (Fig. S3b and d). Notably, for the 2025 season, genus-level profiles at thresholds 0.5 and 0.7 were nearly identical, indicating that major genotype-associated patterns in that season were robust to confidence filtering. Together, these analyses indicate that, while absolute genus composition is sensitive to classification stringency, the overall conclusions regarding genotype- and year-associated variation in dominant bacterial taxa are consistent across confidence thresholds.

In genus-level profiles, fungal reads represented only a minor fraction of total microbial reads, consistent with lower taxonomic richness in fungi than in bacteria. Correspondingly, the number of fungal genera in each sample was substantially lower than that of the bacterial genera. Among fungi, only *Fusarium*, *Aspergillus*, *Cryptococcus*, and *Kluyveromyces* were consistently observed. Of these, *Fusarium* dominated, contributing an average of 92.4% and 79.6% of fungal reads in 2024 and 2025, respectively (Fig. 1e and f). As shotgun metagenomics quantifies DNA, the disproportionately low fungal read counts and genus richness support the interpretation that fungal propagules are relatively less prevalent than bacteria in rhizosphere samples [39, 40].

To compare compositional shifts at the species level, we performed two complementary WMS analyses that identified genera with high internal diversity and strong changes in abundance. This approach revealed the taxa most enriched or depleted relative to the control. Genera such as *Pseudomonas* and *Streptomyces* consistently exhibited high species richness across all barley types, reflecting their widespread association in the rhizosphere. However, the number of species within these genera varied between years. For instance, an average of 23 *Streptomyces* species were detected in 2024, increasing to 71 in 2025, and *Pseudomonas* species increased from 32 to 46 during the same period (Fig. 1g and h). Differential abundance analysis revealed clear contrasts among host genotypes and between years. In both years, less diverse genera exhibited stronger enrichment relative to control soil, highlighting the functional contribution of rare taxa to community composition. Interestingly, plant growth-promoting bacteria such as *Devosia*, *Neorhizobium*, and *Aeromicrobium* were detected predominantly in the rhizosphere of 2024 (Fig. 1i), whereas *Kosakonia* and *Enterobacter* were more abundant in 2025. The modern cultivar Gretchen displayed higher levels of *Erwinia*, *Enterobacter*, and *Tardiphaga* in 2025 (Fig. 1j). These shifts in non-dominant, but functionally relevant taxa indicate variability associated with both host genotype and year-to-year environmental conditions.

In order to compare the shared and unique bacterial genera among barley groups, genus-level data were categorized into four groups including control, wild accessions, landraces, and elite cultivar. A genus was considered present within a category if detected in at least one sample from that group, irrespective of relative abundance. This permissive presence-absence criterion was applied to capture the full detectable taxonomic breadth and to explore shared versus specific-genotype-associated taxa. In both years, a substantial number of taxa were shared across all barley types and the control soil, comprising 104 and 96 genera in 2024 and 2025, respectively (Fig. 1k and l; Additional file 2: Tables. S4 and S5). Despite this common core, distinct sets of genera were unique to each category, indicating genotype- and year-specific associations. Wild accessions from 2024 and landraces from 2025 comprised the highest number of unique genera accounting for 26 each, whereas the elite cultivar Gretchen contained only eight across both years. This pattern indicates differences in the number of unique genera detected across barley groups and years. However, given the limited number of genotypes analyzed, these trends should be interpreted with caution.

Taken together, these results indicate that distinct barley genotypes are associated with characteristic rhizosphere microbiome profiles. While the overall community structure remained conserved across genotypes, consistent with the presence of a shared core microbiome, genotype-dependent differences in relative abundances indicate that host genetic background contributes to variation in rhizosphere community composition, including differential representation of specific bacterial lineages.

### Rhizosphere microbial alpha- and beta-diversity

Alpha and beta diversity analyses were performed on WMS-derived bacterial profiles to assess within (alpha) and between-sample (beta) variation across barley genotypes in 2024 and 2025. Alpha diversity indices, including species richness (Additional file 1: Fig. S4a and d), Shannon (Additional file 1: Fig. S4b and e), and Simpson (Additional file 1: Fig. S4c and f), showed no significant differences among groups (Kruskal–Wallis, *p* > 0.05; Richness: *χ*^2^ = 9.59, *p* = 0.0877 in 2024 and *χ*^2^ = 10.50, *p* = 0.0623 in 2025), indicating comparable overall community diversity. On average, 100–150 bacterial genera were detected in 2024, whereas 2025 samples showed higher richness (220–450 genera) (Additional file 1: Fig. S4a and d), consistent with a more complex rhizosphere community. Wild accessions tended to display slightly higher richness than landraces and the modern cultivar, suggesting modest but non-significant trends consistent with early observations that domestication can narrow rhizosphere diversity [12]. Beta diversity analyses revealed clear genotype- and year-dependent community differences. Principal Coordinate Analysis (PCoA) based on Bray–Curtis and Jaccard distances showed distinct clustering by genotype and year, indicating strong compositional differences, similar to patterns observed in maize and rice [16, 41]. PERMANOVA confirmed significant variation among groups (Bray–Curtis: *R*^*2*^ = 0.79, *F* = 4.64, *p* = 0.001 in 2024, Additional file 1: Fig. S4g; *R*^*2*^ = 0.93, *F* = 16.91, *p* = 0.001 in 2025, Additional file 1: Fig. S4i). In addition, Jaccard-based comparisons supported these findings (*R*^*2*^ = 0.60, *F* = 1.84, *p* = 0.001 in 2024, Additional file 1: Fig. S4h; *R*^*2*^ = 0.56, *F* = 1.54, *p* = 0.001 in 2025, Additional file 1: Fig. S4j), and homogeneity of dispersion tests confirmed that these dissimilarities were not driven by unequal variance among groups. Together, the diversity metrics support genotype-dependent community composition, with stronger divergence observed in 2025, consistent with an additional contribution of seasonal variation.

### Functional profiling of the microbiome reveals conserved yet genotype-specific enrichment of microbial pathways across barley genotypes

To understand whether genotype-dependent community shifts correspond to functional differences, metagenomic assemblies were annotated with eggNOG-mapper to obtain COG and KEGG classifications. COG profiles showed highly consistent functional compositions across genotypes and years, indicating a stable core metabolic structure of the rhizosphere microbiome (Additional file 1: Fig. S5) [42]. The dominant categories across all samples included amino acid transport and metabolism, energy production and conversion, and cell wall-related biogenesis, with ~ 20% of proteins annotated as hypothetical, reflecting the prevalence of uncharacterized microbial functions. Although the total number of predicted proteins varied among genotypes and years, the proportional representation of COG categories remained comparable, suggesting that functional capacities were largely conserved despite taxonomic differences (Additional file 2: Table. S6).

**Fig. 2:**
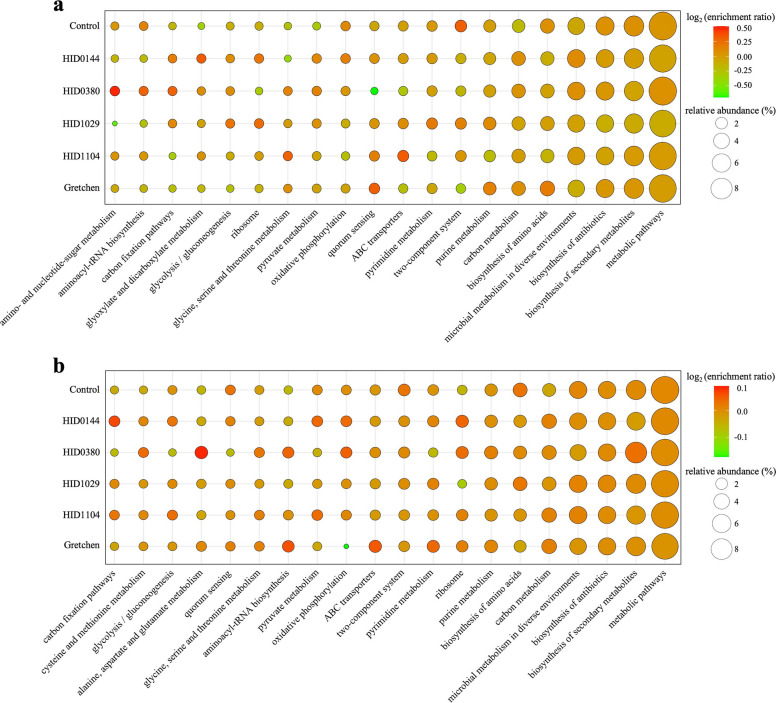
Functional enrichment of KEGG pathways in barley rhizosphere microbiomes from 2024 and 2025. **a, b** Bubble plots showing KEGG pathway enrichment profiles of microbial communities derived from annotated protein datasets in 2024 (**a**) and 2025 (**b**). Each bubble represents an enriched metabolic pathway, with bubble size representing the relative abundance (%) and color indicating the degree of enrichment (green to red gradient). Across both years, core pathways related to amino acid metabolism, energy production, and secondary metabolite biosynthesis were consistently enriched across all genotypes, reflecting conserved metabolic functions in the rhizosphere. Genotype-specific differences were observed, including enhanced enrichment of amino acid and sugar metabolism in wild accessions, and pronounced enrichment of quorum sensing and transport-associated pathways in the modern cultivar Gretchen. The overall higher diversity of enriched pathways in 2025 compared to 2024 indicates functional plasticity within the microbiome across years. Pathway enrichment was assessed using Fischer’s exact test with Benjamini–Hochberg correction. Only pathways with adjusted *p* < 0.05 are shown. Each genotype is represented by two biological replicates

To further understand the specific biochemical pathways involved in the rhizosphere microbial activity, KEGG pathway enrichment analysis was performed on the annotated protein datasets. Across both years, microbial communities from all genotypes exhibited broad enrichment of core metabolic processes, including amino acid metabolism, energy metabolism, and secondary metabolite (SM) biosynthesis (Fig. 2). In 2024 and 2025, an average of 531 and 487, 602 and 348, 555 and 518, and 473 and 379 pathways were enriched in control soil, wild accessions, landraces, and the modern cultivar Gretchen, respectively (Additional file 2: Table. S6). Although most pathways were shared among genotypes, the relative enrichment varied systematically among wild, landrace, and modern types, indicating that differences in microbial composition are accompanied by variation in functional pathway enrichment. In 2024, the wild accession HID0380 displayed strong enrichment in amino acid and sugar metabolism, whereas its closest related counterpart HID0144 showed higher enrichment in glyoxylate and dicarboxylate metabolism (Fig. 2a). The landraces HID1029 and HID1104 exhibited moderate but balanced enrichment across most pathways. Interestingly, HID1029 showed a moderate enrichment in glycolysis or gluconeogenesis, while HID1104 was enriched in ATP-binding cassette (ABC) transporters and amino acid metabolism of glycine, serine, and threonine. The modern cultivar Gretchen displayed a strong enrichment in quorum sensing as well as amino acid biosynthesis, suggesting that microbial communities in its rhizosphere may be more specialized in signaling and metabolic exchange [43]. The dataset from 2025 revealed distinct differences in functional enrichment patterns (Fig. 2b), with an overall higher diversity of enriched pathways compared with 2024. The wild accession HID0144 showed pronounced enrichment in carbon fixation pathways, while HID0380 was enriched in alanine, aspartate, and glutamate metabolism, indicating an increased emphasis on nitrogen assimilation and amino acid turnover. The landraces maintained moderate yet broad enrichment across all major pathways, displaying a similar metabolic profile between HID1029 and HID1104. Interestingly, Gretchen exhibited an altered enrichment pattern relative to its previous year, with elevated activity across aminoacyl-tRNA biosynthesis, ABC transporters, and pyrimidine metabolism, implying enhanced translational and transport processes in its microbiome. Across both years, pathways with antibiotic biosynthesis and SM production were consistently enriched in different genotypes. This pattern points to a metabolically competitive and chemically dynamic rhizosphere environment, where microbial taxa likely engage in both antagonistic and cooperative interactions that shape community structure and potentially contribute to plant protection. Collectively, the functional profiling demonstrates that while core metabolic capacities are conserved across barley genotypes, differential pathway enrichment reveals host-specific microbial adaptation, highlighting associations between host genotypes and microbiome functional profiles.

### Metagenome-assembled genomes (MAGs) reveal the taxonomic structure and recovery quality of the barley rhizosphere microbiome

**Fig. 3:**
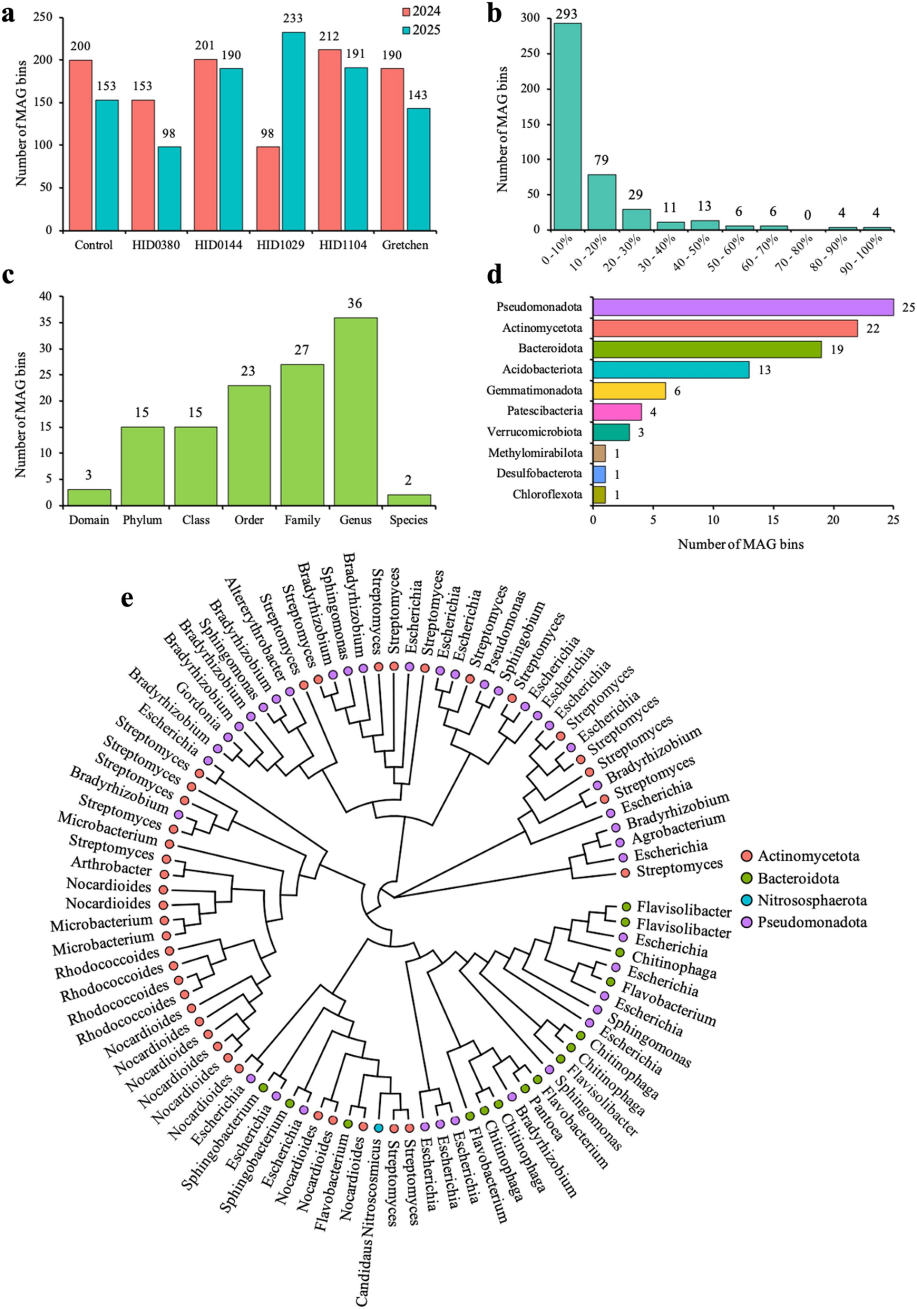
Metagenome-assembled genomes (MAGs) reveal the taxonomic structure and phylogenetic diversity of the barley rhizosphere microbiome. **a** Number of MAG bins reconstructed from metagenomic datasets of barley rhizosphere samples collected in 2024 and 2025, showing variation among genotypes and years. **b** MAG quality assessment based on completeness and contamination estimated by CheckM, indicating that most MAGs exhibited < 50% completeness, consistent with the complexity of soil metagenomes. **c** Taxonomic classification of MAGs at different hierarchical levels using GTDB-Tk, showing the number of MAGs successfully classified at each taxonomic rank. **d** Distribution of MAGs across top 10 bacterial phyla, with *Pseudomonadota*, *Actinomycetota*, *Bacteroidota*, and *Acidobacteriota* comprising the majority of recovered genomes. **e** Phylogenetic cladogram constructed from 16S rRNA genes identified within 93 MAGs, illustrating well-defined clusters corresponding to four major phyla and showing genus-level representatives such as *Bradyrhizobium*, *Chitinophaga*, *Flavobacterium*, *Nocardioides*, and *Streptomyces*

To extend beyond community-level inference and understand genome-resolved diversity, we reconstructed metagenome-assembled genomes (MAGs) from the long-read metagenomic datasets obtained in 2024 and 2025. A total of 445 MAG bins were recovered across all samples, representing genome fragments derived from diverse bacterial lineages (Fig. 3a). The number of reconstructed MAGs varied between samples and years, ranging from 200 and 153 in Control, 153 and 98 in HID0380, 201 and 190 in HID0144, 98 and 233 in HID1029, 212 and 191 in HID1104, and 190 and 143 in Gretchen over 2024 and 2025, respectively. Quality assessment based on CheckM revealed that the majority of MAGs exhibited low completeness (< 50%), with only about 20 bins exceeding this threshold (Fig. 3b). Such fragmentation is characteristic of soil metagenomes, where high species richness, uneven sequencing depth, the intrinsic error profile of nanopore reads, and strain-level complexity limit the recovery of near-complete genomes [44, 45]. Taxonomic classification using GTDB-Tk enabled assignment of MAGs at different ranks, with the number of classified bins increasing from domain to genus level (Fig. 3c). Approximately 15, 23, 27, and 36 distinct phyla, orders, families, and genera were recovered, while only 2 species-level MAGs were identified, reflecting the limited number of high-quality assemblies. A subset of MAGs remained unclassified at lower taxonomic levels, likely due to incomplete marker sets or absence of closely related reference genomes. The most represented bacterial phyla were Pseudomonadota, Actinomycetota, Bacteroidota, and Acidobacteriota, together encompassing the majority of recovered MAGs (Fig. 3d). Among these, Pseudomonadota and Actinomycetota dominated, accounting for 25 and 22 of all bins, respectively. Members of these groups are frequently associated with plant-associated microbiomes and include metabolically versatile taxa capable of stress adaptation, SM production, and organic matter turnover [46–48]. To further resolve evolutionary relationships, a phylogenetic cladogram was constructed using the 16S rRNA genes identified within 93 MAGs (Fig. 3e). The 16S-based tree revealed well-defined clusters corresponding to the four dominant phyla, confirming taxonomic assignments and illustrating the genomic diversity within each lineage. Genera such as *Bradyrhizobium*, *Chitinophaga*, *Flavobacterium*, *Nocardioides*, and *Streptomyces* were among the most represented. The close phylogenetic grouping of these taxa underscores their ecological coherence and prevalence within the barley rhizosphere. Collectively, these results demonstrate that genome-resolved metagenomics enabled recovery and taxonomic placement of bacterial genomes from complex rhizosphere communities, highlighting the predominance of Actinomycetota, Pseudomonadota, and Bacteroidota lineages and revealing fine-scale phylogenetic structure through 16S-based analysis.

### Transcriptional response of barley roots to distinct microbiomes

In order to investigate the genotype-specific transcriptional response of *H. vulgare* roots to distinct microbiome compositions, total mRNA was isolated from root tissues of five barley genotypes in 2025, including wild accessions HID0144 and HID0380, landraces HID1029 and HID1104, and modern cultivar Gretchen. As a control, the high yielding cultivar Gretchen was grown under greenhouse conditions in pots containing sterilized field soil to exclude microbial influence. Two independent biological replicates were analyzed for each genotype, yielding 12 libraries. The ONT sequencing generated approx. 12 million raw reads, of which 10.8 million high-quality reads were retained after trimming. On average, 90% of these clean reads were successfully mapped to the reference genome of *H. vulgare* Morex v3, corresponding to a total of 35,106 genes. Differentially expressed genes (DEGs) were identified using a Benjamini–Hochberg adjusted *p* < 0.05 and a fold change (FC) cutoff of > 2 for increased and < 0.5 for decreased transcript abundances. Given the limited number of biological replicates (*n* = 2), transcriptomic results were interpreted conservatively, with emphasis on reproducible genotype-associated patterns and functional category enrichment rather than marginal gene-level effects.

**Fig. 4:**
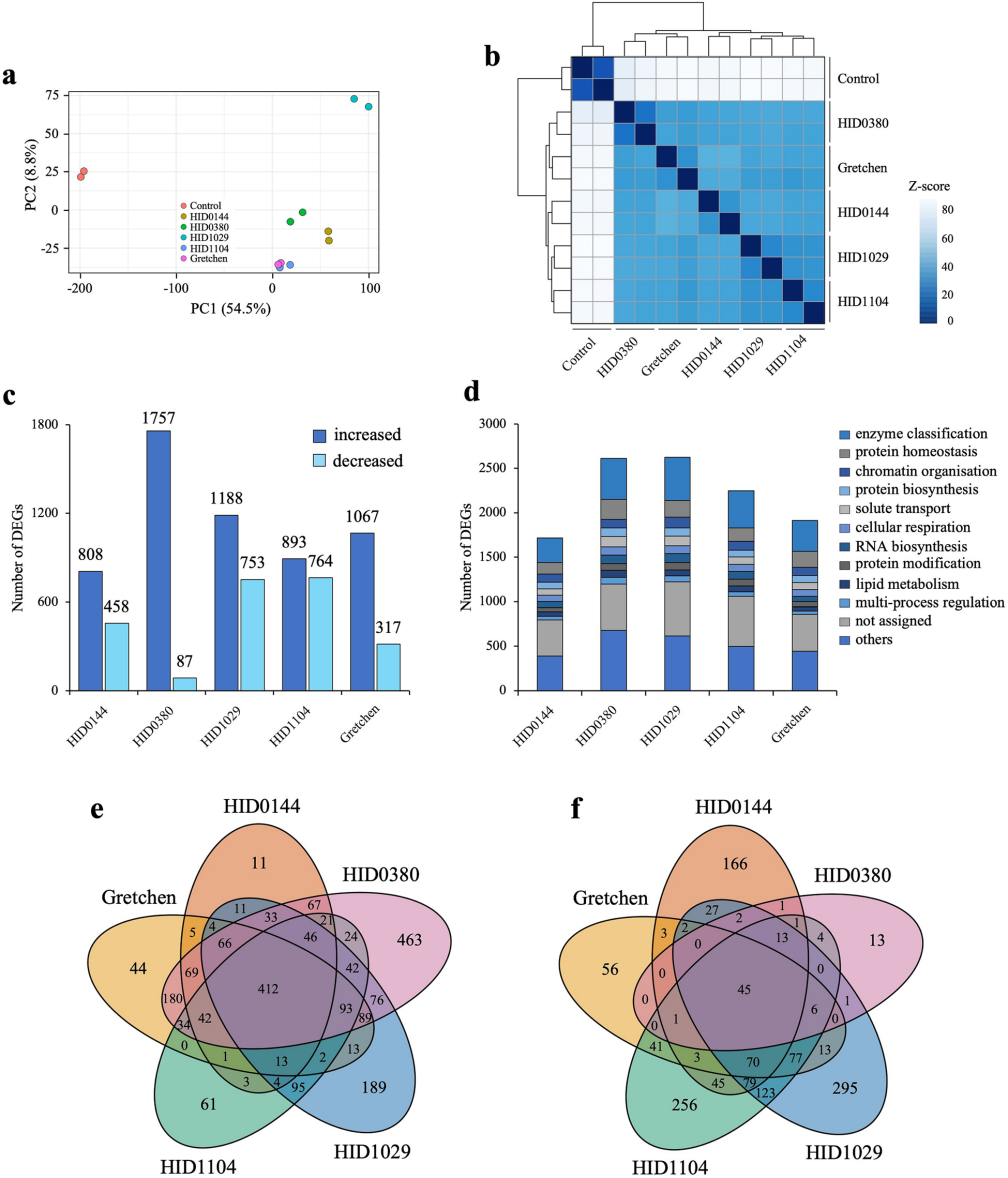
Transcriptome profiling reveals genotype-dependent transcriptional responses of barley roots to distinct microbiome compositions. **a** Principal component analysis (PCA) showed clear clustering of biological replicates and distinct separation among genotypes, indicating strong genotype-specific transcriptional signatures. **b** Heatmap of pairwise sample-to-sample distance matrix based on normalized expression values, confirming high intra-group similarity and inter-genotype divergence. The color code indicates the distance between the samples, as based on the *Z*-score. Dark blue denotes shorter distance, i.e., replicates are grouped closer in distance. **c** Bar chart showing the number of up- (blue) and downregulated (light blue) differentially expressed genes (DEGs) across barley genotypes (adjusted *p* < 0.05, FC > 2 or < 0.5). **d** Stacked bar plot depicting functional classification of DEGs based on Mercator annotation, highlighting predominant categories related to enzyme functions, chromatin organization, RNA biosynthesis, and translational processes. **e, f** Venn diagrams showing the distribution of unique and shared upregulated (**e**) and downregulated (**f**) DEGs among barley genotypes. The number of shared upregulated genes was approximately tenfold higher than shared downregulated genes, reflecting the activation of distinct but overlapping transcriptional programs in response to the rhizosphere microbiome. Each genotype is represented by two biological replicates

Principal component analyses (PCA) showed tight clustering of biological replicates within each genotype, indicating uniformity and high reproducibility of transcriptome profiles, whereas clear separation among genotypes reflected distinct transcriptional signatures (Fig. 4a). This pattern was further supported by a heatmap of the correlation matrices, with samples clustering based on normalized transcript counts (Fig. 4b). The number of DEGs varied among genotypes with 1266, 1844, 1941, 1657, and 1384 DEGs identified in HID0144, HID0380, HID1029, HID1104, and Gretchen, respectively. Of the 1266 and 1844 DEGs of the wild accessions HID0144 and HID0380, 808 and 1757 genes showed increased and 458 and 87 exhibited decreased transcript abundances, respectively. Interestingly, HID0380 had more than twice the number of transcriptionally induced upregulated genes compared with HID0144. The landraces HID1029 and HID1104 showed 1188 and 893 genes with increased expression and 753 and 764 genes with reduced expression, respectively. In the modern cultivar Gretchen, 1067 genes were up- and 317 were downregulated (Fig. 4c). The in-detail summary of the DEGs in all samples is provided in the Additional file 2: Tables. S7—S11. DEGs were functionally categorized using Mercator, revealing strong representation of enzyme-related functions, followed by categories linked to chromatin organization and RNA biosynthesis. Several DEGs were also assigned to functional groups involved in translational processes, including protein homeostasis, biosynthesis, and modification (Fig. 4d). Intriguingly, the Venn plot illustrated genotype-specific transcriptional signatures, identifying 11, 463, 189, 61, and 44 unique upregulated genes and 166, 13, 295, 256, and 56 unique downregulated genes in HID0144, HID0380, HID1029, HID1104, and Gretchen, respectively (Fig. 4e and f; Additional file 2: Tables. S12 and S13). Notably, the number of shared upregulated genes across genotypes was approx. tenfold higher than shared downregulated genes. These results indicate that barley genotypes activate distinct transcriptional responses towards different microbial consortia, highlighting the ability of individual genotypes to respond distinctively to varying microbiome compositions. This correspondence indicates an association between microbiome composition and host transcriptional responses.

To assess functional patterns among differentially expressed genes, gene ontology (GO) enrichment analysis was performed using the biological process (GO:BP) category. The top enriched terms revealed both shared and genotype-specific responses of barley roots to distinct microbiomes (Additional file 1: Fig. S6; Additional file 2: Table. S14). Core processes such as response to stress, small-molecule metabolism, regulation of respiration, and translation-associated functions were consistently enriched across all genotypes. Genotype-specific differences were also evident as HID0144 showed enrichment in nucleoside phosphate metabolism, HID0380 and Gretchen were enriched in catabolic processes, and the landraces displayed similar but quantitatively variable enrichment profiles. The elite cultivar Gretchen additionally showed enrichment related to nucleosome organization, suggesting chromatin-associated regulation. Overall, while major biological processes were conserved, variation in pathway enrichment reflects subtle yet distinct transcriptional programs shaped by genotype and associated microbiomes.

### Host transcriptional activation coordinates with microbial signaling in the barley rhizosphere

**Fig. 5:**
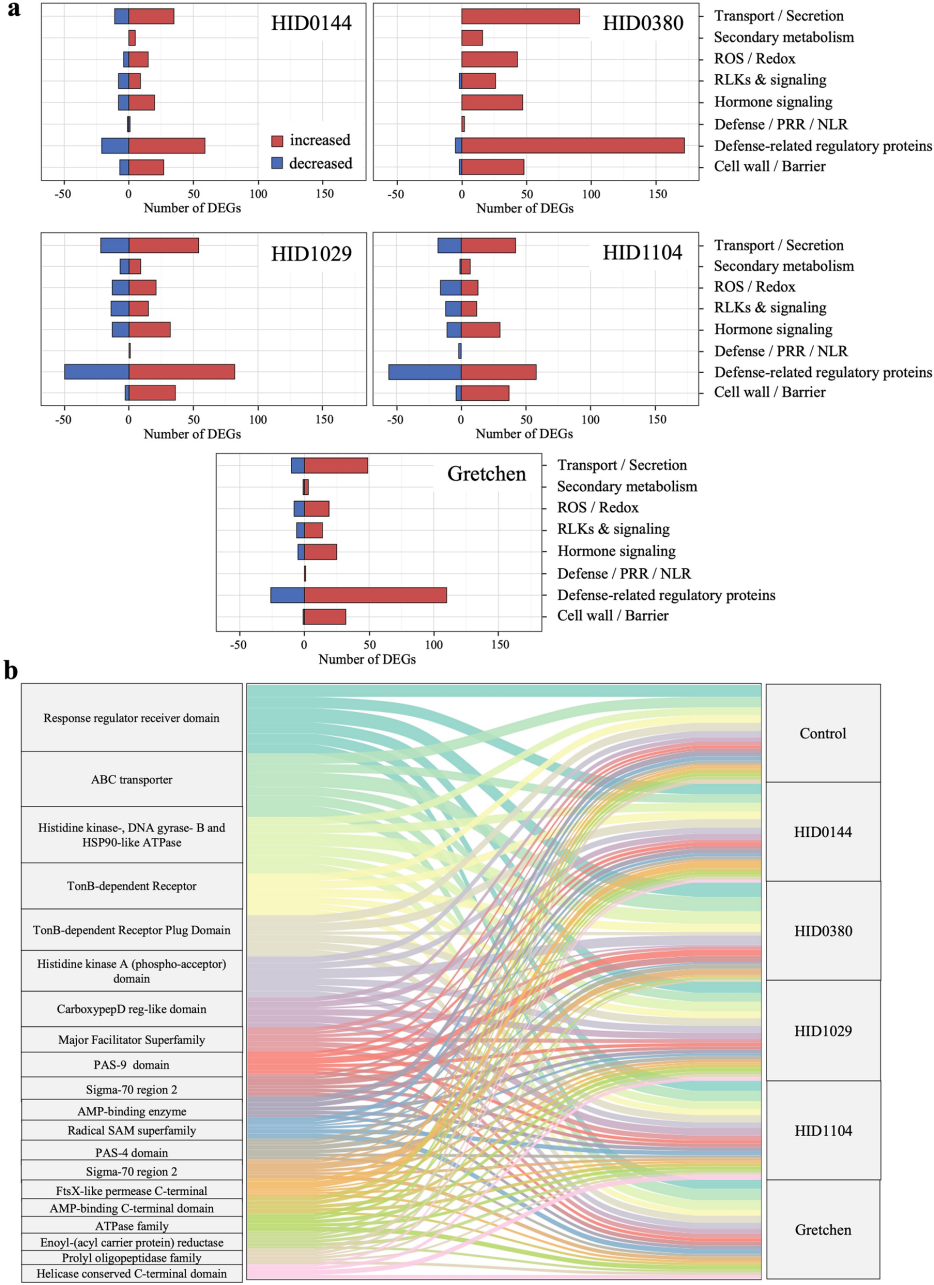
Coordinated transcriptional activation of barley roots and microbial signaling in the rhizosphere. **a** Bar charts showing the number of up- (red) and downregulated (blue) DEGs across eight biological categories relevant to plant–microbe interactions, including transporters, secondary metabolism, redox reactions, receptor-like kinases (RLKs), pattern recognition receptors (PRRs) and nucleotide-binding leucine-rich repeat receptors (NLRs), hormone signaling, defense-related regulatory proteins, and cell wall organization. Across all genotypes, most categories exhibited a predominance of upregulated DEGs, reflecting strong transcriptional activation in response to the rhizosphere microbiome. The wild accession HID0380 displayed the highest transcriptional activation, particularly within defense-related regulatory proteins and transporter categories. **b** Sankey plot illustrating the distribution of dominant Pfam protein families identified in rhizosphere metagenomes from 2025 across the control soil and barley genotypes. Each ribbon represents a Pfam domain linked to one or more samples, with thickness corresponding to the number of detected proteins. The most abundant domains included response regulators, histidine kinases, ABC transporters, and major facilitator superfamily proteins, which are involved in microbial signal transduction and substrate transport. Only Pfam domains with a frequency of five or more occurrences per sample were visualized to highlight the most abundant functional categories

In order to investigate the transcriptional response of barley roots to distinct microbial communities, DEGs were classified into eight biological categories based on Mercator annotation. These categories were selected for their relevance to plant–microbe interactions, and included transporters, secondary metabolism, redox reactions, receptor-like protein kinases (RLKs), hormone signaling, pattern recognition receptors (PRRs) and nucleotide-binding leucine-rich repeat receptors (NLRs), defense-related regulatory proteins, and cell wall organization (Fig. 5a; Additional file 2: Table. S15). Overall, all genotypes exhibited a higher proportion of upregulated genes across most categories, indicating an active transcriptional reprogramming in response to the microbiome. Among the wild accessions, HID0144 and HID0380 showed 171 and 445 DEGs with increased, and 60 and 9 DEGs with decreased transcript abundance, respectively. Notably, HID0380 displayed the strongest transcriptional activation, with 172 and 91 upregulated genes belonging to defense-related regulatory proteins and transporters, respectively. In contrast, only 5 and no downregulated genes were detected in the same category. This highlights the presence of pronounced transcriptional activation in HID0380 taking place in response to the associated microbiota. The landraces HID1029 and HID1104 displayed comparable enrichment patterns, with 250 and 199 upregulated DEGs, respectively. Both genotypes showed a substantial number of regulatory proteins associated with defense, with 82 and 58 DEGs with increased, and 50 and 56 with decreased transcript abundance. The modern cultivar Gretchen showed a total of 253 and 57 up- and downregulated DEGs, including approx. 110 genes with increased activity belonging to the defense-related regulatory proteins category. Across all genotypes, other plant immunity-related groups including PRRs/NLRs, RLKs, and secondary metabolism also contained a substantial number of DEGs, though their representation varied among genotypes.

As the different plant gene categories associated with microbial interactions and signaling showed pronounced transcriptional activation, we decided to look into the protein families within the rhizosphere microbiome from 2025 to understand how microbial communities may respond to host-derived cues. The Pfam-based analysis revealed a conserved set of protein families broadly represented across all samples, suggesting a shared molecular repertoire underlying microbial functionality in the barley rhizosphere. Pfam-based domain annotation identified a total of 537, 682, 62, 4008, 543, and 212 functional domains across control, HID0144, HID0380, HID1029, HID1104, and Gretchen, respectively (Additional file 2: Table. S16). These values represent the total number of predicted protein sequences annotated with a Pfam family within each sample and therefore reflect differences in sequencing depth, assembly size, and predicted proteome complexity rather than direct functional enrichment. Accordingly, downstream analyses focused on relative domain composition and proportional representation of functional categories rather than absolute Pfam counts. The most abundant domains included response regulators, histidine kinases, ABC transporter, and major facilitator superfamily proteins, which are key components of bacterial signal transduction and substrate transport (Fig. 5b) [49, 50]. Their enrichment across all barley genotypes is consistent with a broad capacity for signal transduction and substrate transport in rhizosphere-associated microbes. Interestingly, domains related to redox regulation, including PAS (Per-Arnt-Sim) domain and radical SAM (S-adenosyl-L-methionine) superfamily members, as well as energy-driven transport processes such as ATPases and enoyl-acyl carrier protein reductases, were also highly represented, indicating the prevalence of metabolic and regulatory function in the rhizosphere microbiome.

### Genomic variation at a pathogen defense-related locus reveals structural divergence among barley genotypes

Given the strong transcriptional activation of defense-related regulatory proteins across genotypes (Fig. 5a), we further examined whether variation in the underlying genomic loci could provide additional context for these responses. Among the eight functional categories associated with plant–microbe interactions, this category consistently contained the highest number of upregulated genes in all genotypes. UniProt annotation of 21 commonly induced defense-related genes revealed enrichment of protease inhibitors, ubiquitin–proteasome components, and molecular chaperones such as BAG-domain proteins and calreticulin, classes of proteins that are often associated with plant-pathogen interactions [51–53]. Notably, two of the most highly expressed genes, HORVU.MOREX.PROJ.1HG00003980.1 (BPGv2) and HORVU.MOREX.PROJ.1HG00011330.1 (BPGv2), encode subtilisin/chymotrypsin inhibitor 2 A proteins, typically induced during pathogen challenge and central to protease-mediated immunity [54]. Because these genes showed defense-related transcriptional response across all genotypes, we investigated the genomic structural variation at these loci. Examination of the barley pangenome (BPGv2), together with ONT genome sequencing of our five genotypes, revealed that the two inhibitor genes are paralogs and vary in copy number. PanBarlex (https://panbarlex.ipk-gatersleben.de/) [8] analysis showed that the barley reference Morex carried two paralogous copies, while the wild accession HID0380 contains 16 homologous gene copies within a ~ 600-Kbp cluster, indicating substantial expansion of this defense-associated locus.

To quantify structural divergence in more detail, we applied alignment-free *k*-mer profiling using GeneToCN [55]. The statistical summary of ONT genomic sequencing is provided in Additional file 2: Table. S17. Of the two target genes, only HORVU.MOREX.PROJ.1HG00003980.1 provided sufficient informative k-mers for reliable analysis, and we therefore focused on this locus. Median gene-region *k*-mer depth varied strongly among genotypes, with HID0380 showing the highest depth, consistent with multiple Morex-like copies, whereas HID0144 showed near-zero depth, suggesting absence or extreme divergence of the Morex allele (Additional file 1: Fig. S7a). Other genotypes displayed low to moderate similarity to the reference. To distinguish divergence within the gene from that of the surrounding locus, we separated 25-mers matching the Morex coding sequence (gene-specific) from those derived from the flanking region (flank-specific). All genotypes except HID0144 retained all 30 gene-specific 25-mers, indicating that the coding region itself is structurally conserved. In contrast, flank-specific *k*-mers showed substantial variation with HID1029 retaining 1202 k-mers, indicating highest similarity, whereas HID0380, HID1104, and Gretchen retaining only 12–26 such *k*-mers (Additional file 1: Fig. S7b). Pairwise comparisons of locus-wide *k*-mer sets and hierarchical clustering further resolved these relationships (Additional file 1: Fig. S7c and d). HID0380 and HID1029 formed the closest pair, HID1104 and Gretchen displayed intermediate similarity, while HID0144 was clearly separated from other genotypes. Spatial visualization of *k*-mer abundance reflected the depth of Morex-derived 25-mers at each genomic position across the 30 gene-specific *k*-mers, thereby indicating the relative dosage of Morex-like sequence. This visualization revealed a sharp coverage peak at the gene region for HID0380, reaching a maximum depth of 29, whereas HID1029, HID1104, and Gretchen showed progressively lower depths of 4, 9, and 10, respectively (Additional file 1: Fig. S7e). To determine whether this structural variation was accompanied by nucleotide-level divergence, we performed read-based SNP calling across HORVU.MOREX.PROJ.1HG00003980.1 (Additional file 2: Table. S18). SNP frequencies varied among genotypes, with HID0380 showing the highest number (6 SNPs) that resolved into at least two distinct haplotypes, suggesting potential sub-functionalization among its duplicated copies. The landrace HID1104 carried 4 SNPs, whereas HID0144 and HID1029 each harbored 3 SNPs, and Gretchen showed only a single nucleotide substitution (Additional file 1: Fig. S7f). To assess the positional distribution of these variants, genotype-specific consensus sequences were aligned to Morex and visualized as a per-base match/SNP profile across the 224-bp region of the gene. This revealed that SNPs were mostly uniformly distributed except for the wild accession HID0380, which showed a slightly variable pattern with base substitutions (Additional file 1: Fig. S7g). Together, these results indicate that, although the subtilisin-chymotrypsin inhibitor gene HORVU.MOREX.PROJ.1HG00003980.1 is structurally conserved across barley genotypes, substantial genotype-specific copy number and sequence variation is present at this locus. This variation provides genomic context for genotype-associated differences in transcript abundance of this gene. However, direct experimental validation of copy number variation would be required to establish causality.

Collectively, our multi-omics approach using long-read nanopore sequencing shows that distinct barley genotypes are associated with differences in both the structure and functional potential of their rhizosphere microbiomes. Genotype-dependent shifts in community composition were accompanied by conserved core metabolic functions with variation in specific pathways indicative of genotype-related functional differences within the microbiome. In parallel, host transcriptional responses involving defense, signaling, and metabolic processes coincided with enrichment of microbial transporter and signaling-related domains, highlighting coordinated host-microbiome responses under field conditions. Together with structural variation observed at a pathogen defense-related locus, these findings link plant genomic variation with transcriptional and microbiome-level patterns. However, given the limited number of genotypes subjected to in-depth metagenomic and transcriptomic analysis, these findings should be interpreted as genotype-associated patterns rather than definitive evidence for domestication-level effects.

## Discussion

The rhizosphere represents one of the most complex microbial habitats, where diverse bacteria, fungi, archaea, and protists coexist and interact with plant roots [14]. These dynamic communities are crucial for nutrient turnover, stress resilience, and disease suppression, collectively contributing to plant fitness [56]. Their composition is strongly shaped by root exudates, soil type, and host genotype, creating highly structured yet variable ecosystems [57, 58]. Despite extensive study, the rhizosphere remains difficult to fully characterize due to its immense diversity, uneven microbial distributions, and frequent horizontal gene transfer, which hinder accurate taxonomic assignment and genome assembly [48, 59]. In our study, long-read nanopore sequencing provided insights into how host genotype shapes both the taxonomical and functional landscape of the rhizosphere microbiome. The platform’s ability to generate extended reads enabled improved assembly continuity [60], capturing dominant lineages such as *Streptomyces*, *Bradyrhizobium*, and *Nocardioides*, taxa known for their contributions to nutrient mobilization and stress tolerance [47]. However, a substantial proportion of reads remained unclassified, emphasizing the still-limited representation of soil genomes in reference databases and the challenges inherent to analyzing complex microbial communities [61, 62]. In addition, fungal community profiles derived from shotgun metagenomics are known to underestimate fungal diversity relative to ITS amplicon sequencing due to lower fungal biomass and incomplete reference databases. Accordingly, the apparent dominance of *Fusarium* in our dataset likely reflects both true ecological prevalence and methodological bias, and additional fungal taxa may be underrepresented. Despite these limitations, the Nanopore-based assemblies revealed distinct community structures across genotypes, illustrating how differences in plant genotypes are reflected in microbial composition and genomic complexity.

Genotype-dependent shifts of the barley rhizosphere microbiome represent one of the most important patterns revealed in our study. Across barley genotypes, clear differences in bacterial composition reflect distinct recruitment strategies shaped by domestication and breeding. Similar patterns have been reported in cereals, where wild relatives assemble more diverse and functionally flexible microbiomes than elite cultivars [13]. In barley, host genotype influences community composition through genotype-specific patterns and immune cues [12]. In our study, wild accessions and landrace genotypes harbored broader microbial repertoires, whereas the modern cultivar supported a streamlined but stable community, suggesting a trade-off between resilience and specialization under agricultural selection [22]. In addition, variation in dominant bacterial groups such as Actinomycetota and Pseudomonadota implies genotype-linked modulation of nutrient cycling and stress-associated functions [63]. Preferential enrichment of *Streptomyces* in the modern cultivar and *Bradyrhizobium* in wild genotypes across the years indicates that specific guilds are maintained according to host nutritional and defensive priorities [64]. Together, these observations suggest that barley genotypes are associated with distinct microbial assemblages, consistent with genotype-dependent differences in rhizosphere community composition.

In natural and cultivated systems alike, changes in microbial communities are tightly coupled to functional specialization, as root exudation and immune signaling select for organisms with compatible nutrient and signaling profiles [65]. In our study, across genotypes, metagenomes retained a conserved metabolic backbone dominated by amino acid metabolism, energy generation, and SM production, which are central to survival and plant–microbe communication [66]. Notably, genotype-specific differences appeared in carbohydrate utilization and transport processes, reflecting selective pressures imposed by host-derived carbon inputs. Such variation in metabolic pathways is consistent with reports that plants shape the biochemical landscape of their rhizospheres through differential secretion of sugars, amino acids, and phenolics [58, 67]. These exudates, in turn, guide microbial recruitment and activity, forming feedback loops that sustain nutrient exchange and immune balance [68]. Long-read data further enabled recovery of metagenome-assembled genomes, which highlighted the dominance of Actinomycetota, Pseudomonadota, and Bacteroidota. These taxa are widely recognized inhabitants of plant-associated niches and include metabolically versatile lineages capable of stress adaptation, SM synthesis, and organic matter turnover [69, 70]. Further, genera such as *Chitinophaga*, *Flavobacterium*, *Streptomyces*, and *Sphingomonas* were among the most represented, mirroring patterns commonly observed in rhizosphere communities of cereals and other crops [71, 72]. These taxa likely occupy complementary niches that reinforce rhizosphere stability and enhance plant protection through antibiotic and siderophore biosynthesis [73–76]. Together, these functional and genome-resolved patterns depict a microbiome tuned to host identity, forming a metabolic consortium that co-evolves with barley roots and sets the molecular initiation for coordinated plant–microbe signaling.

The coordination between host roots and the microbiome is rather defined by reciprocal signaling than unilateral responses. In barley roots, the pronounced increase in the transcriptional activation of defense-related genes is consistent with host perception of microbial cues and thereby responding to maintain homeostasis [26]. These gene families are central to the recognition of microbe-associated molecular patterns (MAMPs) and to the establishment of compatibility with beneficial partners [75]. In support of this argument, the rhizosphere microbiome also revealed presence of abundant histidine kinases, response regulators, and transporter-related Pfam domains, which are canonical components of bacterial two-component systems (TCS), thereby consistent with the ability of microbes to sense and respond to plant-derived signals [49]. The concurrent activation of plant perception pathways and microbial signaling systems supports a coordinated host-microbe interaction [77–80]. For instance, in maize, the exudation of sugars, amino acids, and SMs such as benzoxazinoids has been shown to influence rhizosphere assembly and microbial gene expression [68, 79]. In our study, the activation of transporter and SM genes suggests that barley roots modulate chemical exchange, while microbial enrichment in ABC transporters and redox-active domains implies adaptive response to plant-derived chemicals. This reciprocal exchange highlights a coordinated signaling system that maintains metabolic balance and ecological stability at the root-soil interface [80]. The genotype-dependent variation in these interactions further underscores that domestication has shaped the complexity of plant–microbe communication in the rhizosphere.

Wild and landrace genotypes exhibited broader transcriptional activation of redox and signaling genes, aligning with their richer and more flexible microbiomes, whereas the modern cultivar Gretchen favored selective programs that emphasize metabolic regulation and moderated defense. Similar patterns of genotype-dependent microbiome shifts were observed in wheat and maize, where ancestral accessions recruited taxonomically richer microbial communities that stimulated stronger immune and signaling responses [13, 41]. These findings support the hypothesis that breeding has streamlined microbial diversity while selecting associations that preserve core functions with greater stability in cultivated environments [81].

At the transcriptional level, enrichment of defense and hormone signaling-related DEGs supports an integrated regulation of immunity and metabolism in response to microbial presence. In addition, the increased expression of genes belonging to redox and cell-wall-related processes reflects the idea of microbe colonization and biofilm formation along the root surface [82]. On the microbial side, enrichment of radical SAM and PAS domains indicates broad redox and sensory signaling capacities that enable microbes to perceive chemical fluctuations in the rhizosphere, potentially shaping association with plant roots. For instance, the PAS-domain chemoreceptor PcpI in *Pseudomonas* senses plant hormones such as salicylic and indole-3-acetic acid (IAA) and drives chemotactic response to these cues [83]. Together, these host and microbial-related processes point towards a chemical dialog driven by multiple signaling processes. This coordination likely extends also to the regulation of SMs, which function as chemical messengers in plant–microbe interactions. The observed transcriptional activation of SM genes in barley coincided with microbial genomic signatures for non-ribosomal peptide synthetases and polyketide biosynthesis pathways frequently linked to antimicrobial and siderophore production [84, 85]. This is also supported by the enrichment of SM and antibiotic pathways in KEGG from our study. Such coordinated enrichment of chemical pathways is consistent with the concept of “defensive mutualism”, in which microbial metabolites contribute to plant immunity while the host provides the substrates and microenvironments required for microbial SM biosynthesis [86]. For instance, *Pseudomonas fluorescens* producing the polyketide 2,4-diacetylphloroglucinol (2,4-DAPG) was shown to induce systemic resistance (ISR) in *Arabidopsis thaliana*, with mutants deficient in this metabolite losing their ISR-eliciting ability [87]. Similarly, volatiles such as 2,3-butanediol emitted by *Bacillus subtilis* and related rhizobacteria have been demonstrated to trigger systemic immune responses and enhance pathogen resistance in *Arabidopsis* [88]. This parallel enrichment of secondary metabolism genes in both host and microbiome thus reflects a co-adaptive chemical exchange shaping the functional ecology of the rhizosphere. Interestingly, our transcriptional data also highlighted the active participation of transporters and receptor kinases, which may represent molecular gateways through which barley roots respond to microbial signals. In plants, members of the ABC subfamily B (ABCB) have been shown to be essential for arbuscular mycorrhizal symbiosis, facilitating lipid and SM transfer across the peri-arbuscular membrane, thereby sustaining mutualistic nutrient exchange [89, 90]. Similarly, ABCG and ABCC transporters are known to translocate phytohormones, antimicrobial compounds, and signaling molecules, linking transport activity with systemic defense and rhizosphere communication. In case of bacteria, ABC transporters often function as high-affinity importers for amino acids, oligopeptides, and siderophores, serving as key mediators of nutrient acquisition and environmental sensing [91, 92]. Thus, these findings indicate coordinated, two-directional trafficking of small molecules that enables nutrient sharing and signal flow across the root-soil boundary.

Against this background of transcriptional and microbial community variation, plant genomic diversity provides an additional mechanistic layer for understanding genotype-specific microbiome responses. For instance, studies in *Arabidopsis* and maize have shown that natural variation in host loci is associated with shifts in the abundance of key microbial taxa, leading to heritable differences in rhizosphere community structure [41, 93]. Furthermore, SNPs in host immune genes can act as major determinants of microbiome composition, supporting an association between host defense-related loci and microbial assembly [94]. Consistent with this, a GWAS analysis across 200 sorghum genotypes demonstrated that plant genetic variation significantly shapes rhizosphere communities, with distinct loci predicting the abundance of microbial lineages, indicating direct genetic control over microbiome assembly [95]. In our study, the structural variation we observed at a subtilisin-chymotrypsin inhibitor locus, together with its strong transcriptional activation, suggests that defense-related genomic regions may be associated with genotype-specific variation in root-associated microbiomes.

Taken together, the integration of rhizosphere metagenomics, barley root transcriptomics, and plant genomics supports a model in which plant and microbial partners exhibit coordinated patterns of interaction. The concurrent enrichment of signaling, transport, and secondary-metabolic functions across domains shows that the microbiome exhibits signaling capacities associated with host physiological responses. This dynamic interaction, evident in both wild and domesticated genotypes, is consistent with a fine-tuned evolutionary partnership supporting rhizosphere resilience, nutrient exchange, and adaptive stress tolerance, offering actionable entry points for breeding and microbiome-informed crop management.

## Conclusions

Domestication has been accompanied by changes in the genetic architecture of barley that are associated with variation in rhizosphere microbial communities. Using a multi-omics framework based on Oxford Nanopore long-read sequencing, this study shows that the barley rhizosphere microbiome is taxonomically conserved yet functionally variable across wild, landrace, and modern genotypes. The bacterial community, dominated by the phyla Pseudomonadota and Actinomycetota and by genera such as *Streptomyces*, *Bradyrhizobium*, and *Nocardioides*, constituted a shared core microbiome, with relative abundances varying among genotypes and between growing seasons. Functional annotation of metagenomic assemblies revealed a conserved metabolic backbone encompassing amino acid metabolism, energy production, and SM biosynthesis, alongside genotype-associated variation in carbohydrate metabolism and transport pathways. Genome-resolved analyses further provided insight into dominant bacterial lineages, with *Chitinophaga*, *Flavobacterium*, *Streptomyces*, and *Sphingomonas* emerging as key contributors to carbon turnover, secondary metabolism, and root-associated processes. At the host level, transcriptome profiling indicated that barley roots undergo transcriptional reprogramming in response to distinct microbiome compositions, particularly in defense, signaling, and transport processes. These host-associated transcriptional patterns coincided with enrichment of microbial Pfam domains linked to signal transduction and transport, suggesting coordinated functional responses between host and microbiome.

Together, these findings indicate that barley genotype is associated with reproducible differences in the structural and functional potential of the rhizosphere microbiome, and that host and microbial communities exhibit linked molecular responses shaped within the context of domestication and environmental variation. By integrating long-read metagenomics, host transcriptomics and crop genomics, this work provides a framework for exploring genotype-microbiome associations and highlights opportunities for future studies aimed at leveraging plant-microbiome interactions in barley improvement and sustainable crop management.

## Methods

### Plant material and field experimental design

A total of 21 barley genotypes were included in the field experiment, comprising seven wild accessions, seven landraces, and seven modern cultivars selected to represent a broad domestication gradient. Genotypes were selected based on their variety type (“spring”) from a larger diversity panel of 64 accessions (39 wild accessions, 8 landraces and 17 elites), that are continuously grown and phenotypically screened over multiple years (experiment started in 2020). All genotypes were grown concurrently under identical field conditions in micro plots (*N*_total_ = 30, distributed in three rows with 10 plants per row) at the experimental field station of Martin Luther University Halle-Wittenberg (“Kühnfeld”, 51° 29′ 46.47″ N; 11° 59′ 41.81″ E) to enable comparative field and rhizosphere microbiome analyses. Soil sampling from the root rhizosphere was done in 2024 and 2025. Field plots were managed following standard agronomic practices, but omitting pesticides and herbicides. Detailed information on geology, soil characteristics, and climatic conditions of the site has been reported previously [96, 97]. Briefly, the soil at the site is classified as a slightly degraded Haplic Phaeozem developed on sandy loess, with a texture consisting of approximately 55% sand, 33% silt, and 12% clay. The soil profile extends to a depth of ~ 0.8 m and overlays glacial till, with the groundwater level located at approximately 1.5 m depth.

Rhizosphere samples from this field experiment were initially profiled to assess microbiome variation across the full genotype set. Based on these profiles, five representative genotypes spanning wild, landrace, and modern cultivar categories were selected for in-depth long-read metagenomic sequencing. Importantly, the metagenomic analyses were performed on rhizosphere samples originating from the same field-grown plants used for initial profiling. To assess the reproducibility of genotype-associated microbiome patterns across growing seasons, these five genotypes were subsequently cultivated again in an independent field season at the same experimental site and using the same experimental procedure.

### Collection of soil and plant material

At harvest, rhizosphere soil was defined as the fraction adhering to roots after gentle shaking and was collected with sterile spatulas. All samples were collected at the same developmental stage (8 weeks after sowing) to ensure comparability across genotypes, field seasons, and experimental conditions. The collected material was transferred into sterile bags, transported on dry ice, and stored at − 80 °C until DNA extraction. As a non-treated control, soil was collected from unplanted areas within the same experimental field used for barley cultivation during the corresponding field season(s). This control soil was used as a reference to characterize the background soil microbial community and was not intended for direct genotype-level comparisons.

For transcriptome analyses, root material was harvested from the same plants at the same developmental stage. Roots were cut with sterile scissors, washed several times with sterile water to remove loosely attached soil, blotted dry, and stored at − 80 °C until RNA isolation. In addition, field soil was collected and sterilized by autoclaving and used for plant cultivation under greenhouse conditions. As a control for RNA-Seq, Gretchen plants were grown in sterilized field soil in the greenhouse, and roots were harvested for RNA isolation at 8 weeks.

For genomic DNA (gDNA) sequencing, barley plants were cultivated in the greenhouse at the Institute of Agricultural and Nutritional Sciences (MLU, Halle (Saale), Germany). Leaves were harvested after 4 weeks and approximately 1 g of leaf tissue was collected and stored at − 80 °C until DNA isolation.

For each genotype, two independent biological replicates were sampled per field season. Each biological replicate consisted of three individual soil or roots, which were pooled to minimize within-plot variability. This experimental design was applied consistently across both field seasons.

### Soil DNA isolation and 16S rRNA amplicon sequencing

Soil microbial gDNA extraction for amplicon sequencing was performed using the DNeasy PowerSoil Pro Isolation Kit (Qiagen, Hilden, Germany) according to the manufacturer’s instructions. The concentration of gDNA was quantified using a NanoDrop ND-1000 spectrophotometer (NanoDrop Technologies, Montchanin, DE, USA), and the extracts were adjusted to 20 ng/μL template concentration followed by amplification of the bacterial 16S rRNA gene’s V4 region using the primer pair 515 F (GTGYCAGCMGCCGCGGTAA) and 806R (GGACTACNVGGGTWTCTAAT) [98] with Illumina adapter overhangs. The amplified products were purified with Agencourt AMPure XP beads (Beckmann Coulter, Krefeld, Germany). The amplified fragments were indexed using Illumina Nextera XT indices at both ends through indexing PCR. The indexed products were subsequently purified with AMPure beads, quantified by Quant-iT dsDNA high-sensitivity assay kit using a TECAN INFINITE PLEX plate reader (TECAN, Germany) and sample-based amplicon libraries were equimolarly pooled to achieve a final pool concentration of 4 nM. Finally, paired-end 2 × 300 bp sequencing was performed with a MiSeq Reagent kit v2 on an Illumina MiSeq platform (Illumina Inc., San Diego, CA, USA).

### Soil DNA isolation for whole metagenome sequencing

Soil gDNA for whole metagenome sequencing (WMS) was extracted from rhizosphere soil using a combined mechanical and chemical lysis approach adapted from different soil DNA extraction protocols [99, 100]. Approximately 250 mg of soil was placed in a 2-mL tube containing ~ 200 mg of 0.5-mm glass beads (Roth, Karlsruhe, Germany), followed by the addition of 1 mL of preheated CTAB extraction buffer (2% CTAB, 1.4 M NaCl, 100 mM Tris–HCl pH 8.0, 20 mM EDTA, 0.2 M mannitol, and 2.6% PVP). Samples were homogenized twice at 25 Hz for 2.5 min in a TissueLyser (Qiagen, Haan, Germany), reorienting the adapter between runs, and incubated at 65 °C for 30 min with periodic inversion. Cell debris was pelleted by centrifugation at 14,000 × *g* for 10 min at 4 °C, and the supernatant was subjected to phenol: chloroform: isoamyl alcohol (25:24:1) extraction, followed by a second purification with chloroform:isoamyl alcohol (24:1). DNA was precipitated with 0.6 volumes of cold isopropanol and 0.1 volume of 3 M sodium acetate, incubated overnight at − 20 °C, and recovered by centrifugation. Pellets were washed with 70% ethanol, air-dried, and resuspended in 25 µL of nuclease-free water. To remove residual inhibitors, DNA was further purified using the Zymo DNA Clean & Concentrator kit (Zymo Research, Freiburg, Germany). DNA yield was measured with a Qubit fluorometer (Thermo Fisher Scientific, Singapore), and the sample was adjusted to 500 ng for sequencing purposes.

### Isolation of plant genomic DNA for whole genome sequencing

Plant gDNA for whole genome sequencing (WGS) was isolated from 4-week-old barley plants using the NucleoBond HMW DNA Kit (Macherey-Nagel, Düren, Germany) according to the manufacturer’s instructions. Frozen leaf samples were ground to a fine powder and transferred into a 2-mL tube containing two sterile steel beads (Ø 3.2 mm) (Hecht Kugellager GmbH & CoKG, Winnenden, Germany). Samples were homogenized in a TissueLyser at 25 Hz for 30 s and DNA was extracted using the kit protocol. DNA concentration was measured with a Qubit fluorometer, and the sample was adjusted to 400 ng for sequencing.

### Isolation of RNA from barley roots

Total RNA was isolated from barley root tissue using the Nucleospin RNA Plant and Fungi kit (Macherey–Nagel, Düren, Germany) following the manufacturer’s instructions with minor modifications. Frozen root samples stored at − 80 °C were ground to a fine powder in a pre-chilled mortar with liquid nitrogen. Approximately 300 mg of powdered root tissue was transferred into a 2-mL RNase-free tube containing two sterile steel beads (Ø 3.2 mm). Samples were homogenized in a TissueLyser at 30 Hz for 1 min, the adapter was reoriented, and homogenization was repeated to ensure complete disruption. RNA was then extracted using the kit protocol, yield was measured with a Qubit fluorometer, and the concentration was adjusted to 750 ng total RNA for sequencing purposes.

### Oxford long-read nanopore library preparation and sequencing

The gDNA from rhizosphere samples and barley leaves was prepared for nanopore sequencing using the SQK-NBD114.96 and SQK-NBD114.24 native barcoding kit (Oxford Nanopore Technologies (ONT), Oxford, England), respectively, following the manufacturer’s instructions with minor adjustments. A high molecular weight (HMW) DNA standard (Zymo Research, California, USA) was also included as a performance control for WMS to evaluate long-read sequencing efficiency and data quality. DNA repair, end preparation, native barcode, and adapter ligation were carried out according to the kit protocol. Clean up steps were performed using AMPure XP beads and adapter ligation was performed with long fragment buffer (LFB) to retain longer DNA fragments for WMS.

For transcriptome sequencing, libraries were prepared using the cDNA-PCR Barcoding Kit SQK-PCB114.24 (ONT, Oxford, England) according to the manufacturer’s instructions. A total of 750 ng of high-quality RNA was reverse transcribed into cDNA, barcoded, and amplified by PCR for 16 cycles, followed by exonuclease treatment and cleaned up as described in the kit protocol.

All libraries were sequenced on R10.1 flow cells on a PromethION platform for three consecutive days. Libraries were prepared simultaneously and sequencing was continuous, with intermediate flow cell washing and reloading on days 2 and 3 using the Flow Cell Wash Kit EXP-WSH004 (ONT, Oxford, England), without changing flow cells or sequencing parameters. Sequencing was managed using MinKNOW software (ONT), and basecalling was performed with the Dorado super accurate model using a minimum Q-score of 10 for genomic and metagenomic, and 7 for transcriptomic libraries.

### Processing and analysis of Illumina data for 16S study

The sequence data analysis was performed using QIIME2 (v2023.9) [101]. Briefly, forward and reverse primers from demultiplexed reads were trimmed using q2-cutadapt [102] followed by denoising with DADA2 (v1.26) [103], chimera removal and clustering into Amplicon Sequence Variants (ASVs). Taxonomy was assigned to ASVs using the q2‐feature‐classifier [104] sklearn-naïve Bayes taxonomy against the silva-138–99–515–806-nb-classifier reference database [105]. The bacterial phylogenetic tree was inferred using the q2-phylogeny plugin which employs MAFFT (v7.520) [106] for sequence alignment and FastTree (v2.2.0) for Maximum Likelihood tree construction [107]. The tree was midpoint-rooted to ensure a robust and unbiased phylogenetic framework. The ASV matrices, taxonomic tables, phylogenetic tree, and representative sequences were merged with the sample metadata file using the phyloseq package (v1.52.0) [108] in R (v4.5.1) [109]. The bacterial ASV matrices were rarefied to ensure uniform sequencing depth of 30,375 reads per sample. The role of the barley domestication status on the rhizosphere bacterial community compositions (based on Bray–Curtis distance dissimilarity) was analyzed through PERMANOVA using the adonis2 function from the vegan package (v2.7.1) [110], followed by a pairwise adonis test using the pairwise.adonis2 package (v0.4) [111] with Benjamini‐Hochberg false discovery rate (FDR) correction [112]. The ordinations were visualized using Principal coordinates analysis (PCoA) as implemented in the plot ordination function of phyloseq using Bray–Curtis distances.

### Processing and analysis of ONT data for metagenomics

Raw reads generated from Oxford Nanopore sequencing were uploaded to the Galaxy Europe platform (https://usegalaxy.eu/) for downstream analysis. Read quality was assessed with fastplong (v0.4.1) [113], and low-quality reads trimmed with cutadapt (v5.1). Taxonomic assignment of microbial reads was performed with Kraken2 (v2.1.3) [114] using the prebuilt bacterial and fungal genome databases with confidence thresholds of 0.1, 0.5, and 0.7 to ensure the sensitivity of taxonomic profiles to confidence stringency. Kraken2 read counts were summarized at phylum, genus, and species levels and imported into R for analysis. Biological replicates were merged, relative abundances were calculated, and the ten most abundant taxa at each rank were retained. Data handling and visualization were performed with tidyverse package (v2.2.0) [115] and ggplot2 (v4.0.0) [116]. Differential abundance was analyzed with DESeq2 (v1.46.0) [117], using the control as reference and applying apeglm shrinkage (v1.28.0) [118]. Genera with adjusted *p* < 0.05 were visualized using ComplexHeatmap package (v2.22.0) [119]. Shared and unique bacterial genera among control, wild accession, landrace, and modern groups were visualized with VennDiagram package (v1.7.3) [120]. Alpha diversity metrics including species richness, Shannon and Simpson index were calculated with vegan [110] and visualized in ggplot2. Statistical differences were tested using the non-parametric Kruskal–Wallis test [121]. Beta diversity was assessed using Bray–Curtis and Jaccard dissimilarities, followed by principal coordinate analysis (PCoA) in ape (v5.8.1) [122], and visualized in ggplot2.

### Functional profiling of rhizosphere microbiome

Nanopore long-read sequencing metagenomic data were assembled and functionally annotated to understand the functional potential of the rhizosphere microbiome. Briefly, the biological replicates for each sample were merged to improve coverage, and assemblies were generated with metaFlye (v2.9.6) [123]. Open reading frames (ORFs) were predicted with Prodigal (v2.6.3) [124], and protein sequences were annotated using eggNOG-mapper (v2.1.12) [125] with the DIAMOND algorithm and a threshold of 0.001. Annotations included Clusters of Orthologous Genes (COGs), KEGG Orthologs (KOs), pathways, and modules. Functional summaries were generated using pandas (v2.2.2) [126], and KEGG pathways were visualized in R using the tidyverse and ggplot2. Predicted proteins were further screened for Pfam domains (http://pfam.xfam.org/) [127] through eggNOG-mapper, and Pfam frequencies were calculated for each sample. Only domains with at least 5 occurrences were retained for comparison. The top 20 Pfam families were visualized in ggplot2 and ggalluvial (v0.12.5) [128]. Pfam accessions were cross-referenced with their corresponding functional descriptions based on the Pfam-A database (v35.0) for biological interpretation.

### Metagenome-assembled genomes (MAGs) reconstruction and quality assessment

Metagenomic reads from 2024 and 2025 were co-assembled to generate a single composite assembly representing the pangenome of the microbial community. Co-assembly of metagenomic reads across genotypes and field seasons was performed to increase assembly continuity and genome recovery from highly diverse rhizosphere communities with uneven sequencing depth. The concatenated dataset was assembled with metaFlye in nano-hq mode. The resulting contigs were used as a reference for read mapping with minimap2 (v2.28) [129] to estimate per-sample contig coverage, and coverage profiles were summarized as a depth matrix for binning with MetaBAT2 (v2.17) [130]. MAG quality was evaluated with checkM (v1.2.4) [131] by estimating completeness and contamination based on lineage-specific marker genes. Taxonomic assignment was performed using GTDB-Tk (Genome Taxonomy Database Toolkit, v2.5.2) [132], yielding standardized lineage classifications for all reconstructed bins. In addition, 16S rRNA genes were identified using Barrnap (v0.9) [133], aligned with MAFFT and used for phylogenetic reconstruction with FastTree. Data visualization and graphical summaries were performed in R using the ggtree package (v3.10.0) [134].

### Processing of transcriptome data

Raw ONT reads were assessed for quality using fastplong within the galaxy platform. Adapter sequences and low-quality bases were removed with cutadapt and poly A/T stretches of at least 10 bases were trimmed to prevent alignment artifacts and a Phred quality cutoff of 20 was applied to remove low-quality bases. The trimmed reads were aligned to the *Hordeum vulgare* reference genome (https://ftp.ensemblgenomes.ebi.ac.uk/pub/plants/release-62/fasta/hordeum_vulgare/dna/; accessed on October 3, 2025) using Minimap2 in spliced alignment mode optimized for ONT reads (map-ont) with splice junction support from the corresponding gene annotation file (https://ftp.ensemblgenomes.ebi.ac.uk/pub/plants/release-62/gff3/hordeum_vulgare/). Gene level read counts were obtained with featureCounts (v2.1.1) [135], and differential expression analysis was performed with DESeq2 (v2.11.40.8). Genes with an adjusted *p* < 0.05, and a fold change (FC) > 2 or < 0.5 were considered significant for increased or decreased transcript abundance, respectively. Principal component analysis (PCA) and sample-to-sample correlation matrices were generated in DESeq2 and the numbers of unique and shared differentially expressed genes (DEGs) were visualized with the VennDiagram package in R. Given the limited number of biological replicates per genotype (*n* = 2), differential expression analyses were performed conservatively. DESeq2 was selected due to its shrinkage-based dispersion estimation, which stabilizes variance estimates in low-replicate designs. Downstream interpretation focused on robust expression patterns, functional category enrichment, and consistency across genotypes rather than marginal gene-level effects.

### Functional annotation and enrichment analysis of transcriptome

Functional categorization of DEGs was performed with the Mercator annotation platform (https://www.plabipd.de/mercator_main.html) [136], which assigns genes to hierarchical functional bins based on conserved domains and sequence homology. The *H. vulgare* protein reference file (https://ftp.ensemblgenomes.ebi.ac.uk/pub/plants/release-62/fasta/hordeum_vulgare/pep/; accessed on October 7, 2025) was used for annotation. DEGs obtained from the RNA-Seq analysis were mapped to Mercator annotations and merged DEG-annotation tables in R using tidyverse. Genes were grouped by functional category and the ten most represented bins were identified. Gene Ontology enrichment analysis was carried out on significantly expressed genes with Benjamini–Hochberg adjusted *p* < 0.05 with gprofiler2 [137], using *H. vulgare* (Morex v3) genome as reference. Enrichment was calculated against the background of all expressed genes using Fischer's exact test with Benjamini–Hochberg false discovery rate (FDR < 0.05) correction. GO was performed on the Biological Process (BP) category, and the most significant terms were visualized as bubble plots in ggplot2. To investigate transcriptomic responses of barley roots to microbial interactions, DEGs were screened for functional classes associated with categories of defense, signaling, and secondary metabolism using the Mercator/MapMan annotations [138]. Category-based DEG counts were summarized and visualized in ggplot2.

### Processing and analysis of ONT data for plant genomics

Raw ONT WGS reads from all barley genotypes were analyzed using the GeneToCN tool [55], which performs alignment-free copy number estimation based on *k*-mer frequencies. Reference genome sequences of Morex BPGv2 (https://panbarlex.ipk-gatersleben.de/) were used to extract the target gene and its flanking regions, which were processed with the GeneToKmer script to generate 25-bp *k*-mers unique to the reference locus. These gene-specific and locus-wide *k*-mers were queried against genotype-specific ONT reads using GenomeTester4 [139]. For each genotype, *k*-mer frequencies were counted, and the median gene-specific *k*-mer depth was calculated as a proxy for genomic dosage. Locus-wide and gene-specific *k*-mer counts were then summarized to assess sequence similarity and divergence relative to the Morex reference. Pairwise *k*-mer intersections between genotypes were used to compute a similarity matrix, which was visualized as a heatmap and hierarchical clustering dendrogram. Spatial variation in *k*-mer abundance was examined by mapping *k*-mer counts back to genomic coordinates, binning, and smoothing to generate coverage profiles using ggplot2. For the analysis of read-based variant calling to identify SNPs and insertions and deletions (INDELS), the ONT reads were aligned to the Morex reference genome using minimap2. Subsequently, we screened for genetic diversity using clair3 [140] for SNPs as well as sniffles2 [141] for INDELS. Per-base SNPs within the coding region were visualized using ggplot2. All downstream data handling, and figure generation were performed in R.

## Supplementary Information

Additional file 1: Figures S1—S7. Figure S1. Principal Coordinates Analysis (PCoA) of rhizosphere bacterial communities across barley domestication groups. Figure S2. Oxford Nanopore whole-metagenome sequencing (WMS) statistics of rhizosphere microbiome samples from 2024 and 2025. Figure S3. Effect of Kraken2 confidence thresholds on bacterial taxonomic profiles of barley rhizosphere metagenomes. Figure S4. Alpha and beta diversity of WMS-derived bacterial communities in rhizosphere samples from 2024 and 2025. Figure S5. Functional categorization of predicted proteins from metagenomic assemblies across rhizosphere samples in 2024 and 2025. Figure S6. Gene Ontology (GO) enrichment analysis of differentially expressed genes (DEGs) across barley genotypes. Figure S7. Structural and nucleotide-level variation at the HORVU.MOREX.PROJ.1HG00003980 locus across barley genotypes.

Additional file 2: Tables S1—S18. Table S1. Pairwise PERMANOVA results comparing rhizosphere bacterial community composition across barley status groups and controls. Table S2. Summary statistics of whole metagenome sequencing and taxonomic classification for 2024 samples. Table S3. Summary statistics of whole metagenome sequencing and taxonomic classification for 2025 samples. Table S4. Unique and shared bacterial genera among different cultivar categories in 2024 samples. Table S5. Unique and shared bacterial genera among different cultivar categories in 2025 samples. Table S6. Summary statistics of predicted protein annotation and functional classification derived from whole metagenomic assemblies. Table S7. DEGs of the wild accession HID0144 compared with the control. Table S8. DEGs of the wild accession HID0380 compared with the control. Table S9. DEGs of the landrace HID1029 compared with the control. Table S10. DEGs of the landrace HID1104 compared with the control. Table S11. DEGs of the elite cultivar Gretchen compared with the control. Table S12. Unique and shared list of upregulated genes among transcriptomic samples. Table S13. Unique and shared list of downregulated genes among transcriptomic samples. Table S14. Enriched Gene Ontology (GO) biological process (BP) terms identified across different transcriptome samples. Table S15. Plant-specific functional categorization of DEGs during microbial interactions. Table S16. Summary of Pfam protein families identified from metagenomic assemblies across barley rhizosphere samples. Table S17. Summary statistics of whole genome sequencing of barley genotypes. Table S18. Genomic coordinates and sequence variation of the target gene HORVU.MOREX.PROJ.1HG00003980 on chr1H:9,112,788–9,113,012.

## Data Availability

All sequence data generated in this study have been deposited in public repositories. The amplicon-based microbiome profiling data generated by Illumina sequencing are available under NCBI BioProject PRJNA1420646 (https://www.ncbi.nlm.nih.gov/bioproject/PRJNA1420646) [142]. The Oxford Nanopore sequencing datasets generated in this study are available at the European Nucleotide Archive (ENA) under Projects PRJEB107287 (https://www.ebi.ac.uk/ena/browser/view/PRJEB107287) [143], PRJEB107425 (https://www.ebi.ac.uk/ena/browser/view/PRJEB107425) [144], and PRJEB107426 (https://www.ebi.ac.uk/ena/browser/view/PRJEB107426) [145].

## Abbreviations

ABC: ATP-binding cassette
AMF: Arbuscular mycorrhizal fungi
ASV: Amplicon Sequence Variant
BPGv2: Barley Pangenome version2
COG: Cluster of Orthologous Genes
CTAB: Cetyltrimethylammonium Bromide
DEGs: Differentially expressed genes
eQTL: Expression quantitative trait locus
FC: Fold change
FDR: False Discovery Rate
gDNA: Genomic DNA
GO: Gene Ontology
GO:BP: Gene Ontology Biological Process
GWAS: Genome-wide association study
HMW DNA: High molecular weight DNA
ISR: Induced systemic resistance
ITS: Internal Transcribed Spacer
KEGG: Kyoto Encyclopedia of Genes and Genomes
KO: KEGG Orthology
MAGs: Metagenome-assembled genomes
MAMP: Microbe-associated molecular pattern
NLR: Nucleotide-binding leucine-rich repeat receptor
ONT: Oxford Nanopore Technologies
ORF: Open reading frame
PAS: Per-Arnt-Sim
PCA: Principal component analysis
PCR: Polymerase chain reaction
PCoA: Principal Coordinate Analysis
PRR: Pattern recognition receptors
PVP: Polyvinylpyrrolidone
RLK: Receptor-like protein kinases
rRNA: Ribosomal RNA
SAM: S-adenosyl-L-methionine
SM: Secondary metabolite
SNP: Single-nucleotide polymorphism
SV: Structural variation
TCS: Two-component system
WGS: Whole genome sequencing
WMS: Whole metagenome sequencing
