# Supplementary material for: Nanopore sequencing reveals coordinated host and microbiome responses across barley genotypes
Source: BMC Biol. 2026 Jul 29;24:177. doi: 10.1186/s12915-026-02688-3 (PMC13425779; doi:10.1186/s12915-026-02688-3)
Supplement: Supplementary file 1 — Additional file 1: Figures S1—S7. Figure S1. Principal Coordinates Analysis (PCoA) of rhizosphere bacterial communities across barley domestication groups. Figure S2. Oxford Nanopore whole-metagenome sequencing (WMS) statistics of rhizosphere microbiome samples from 2024 and 2025. Figure S3. Effect of Kraken2 confidence thresholds on bacterial taxonomic profiles of barley rhizosphere metagenomes. Figure S4. Alpha and beta diversity of WMS-derived bacterial communities in rhizosphere samples from 2024 and 2025. Figure S5. Functional categorization of predicted proteins from metagenomic assemblies across rhizosphere samples in 2024 and 2025. Figure S6. Gene Ontology (GO) enrichment analysis of differentially expressed genes (DEGs) across barley genotypes. Figure S7. Structural and nucleotide-level variation at the HORVU.MOREX.PROJ.1HG00003980 locus across barley genotypes. [file 12915_2026_2688_MOESM1_ESM.docx]

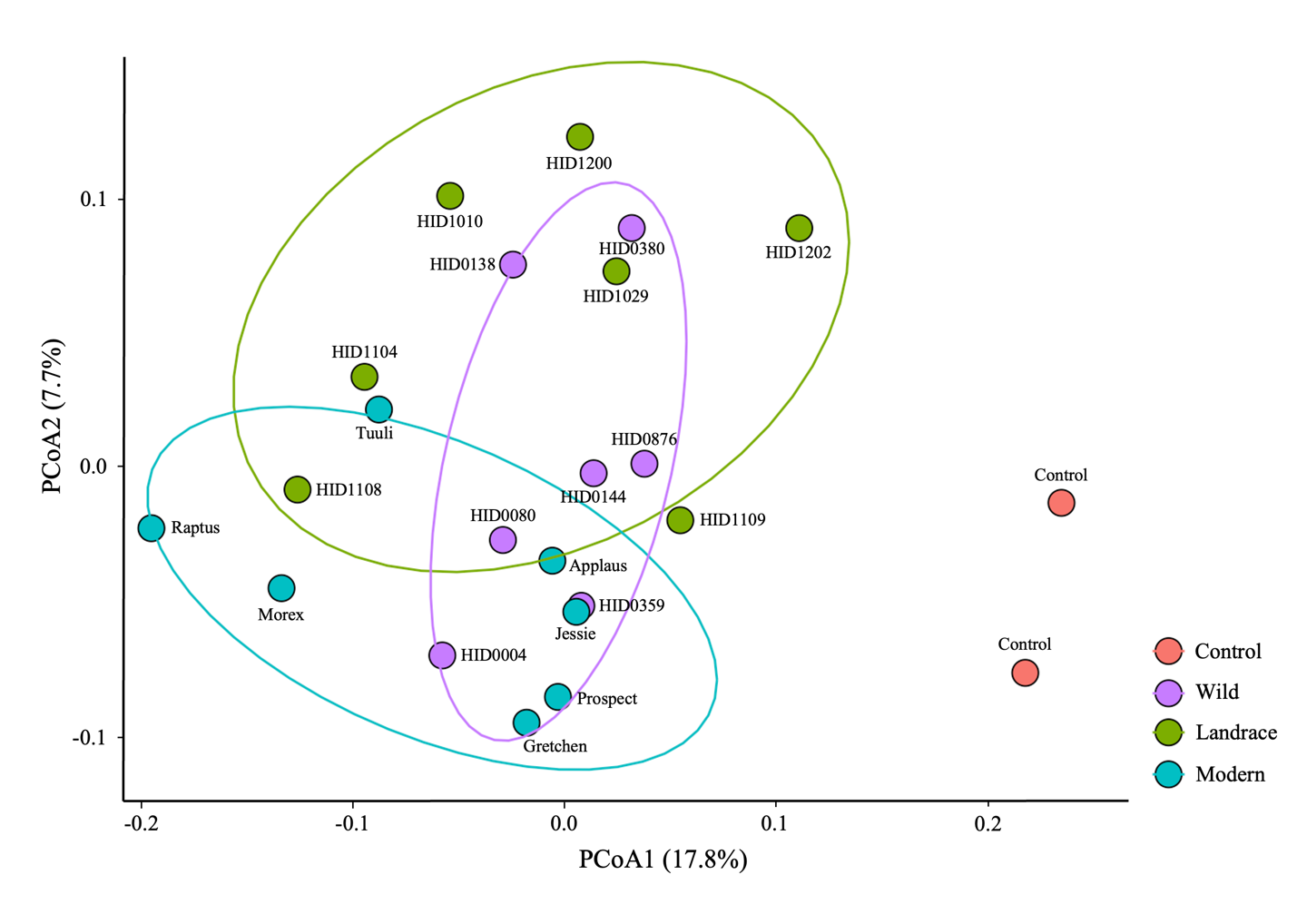


# **Figure S1. Principal Coordinates Analysis (PCoA) of rhizosphere bacterial communities across barley domestication groups.**

PCoA based on Bray-Curtis dissimilarities of bacterial ASV profiles derived from 16S rRNA gene amplicon sequencing of 23 barley genotypes representing four domestication groups including control (red), wild (purple), landrace (green), and modern (blue). Samples were transformed to relative abundances prior to ordination, and 70% confidence ellipses were drawn around each group to illustrate multivariate dispersion. Distinct clustering patterns reflect differences in rhizosphere bacterial community composition associated with domestication status. Genotype labels are displayed for each point. Each data point represents one sample (n = 2 per genotype).


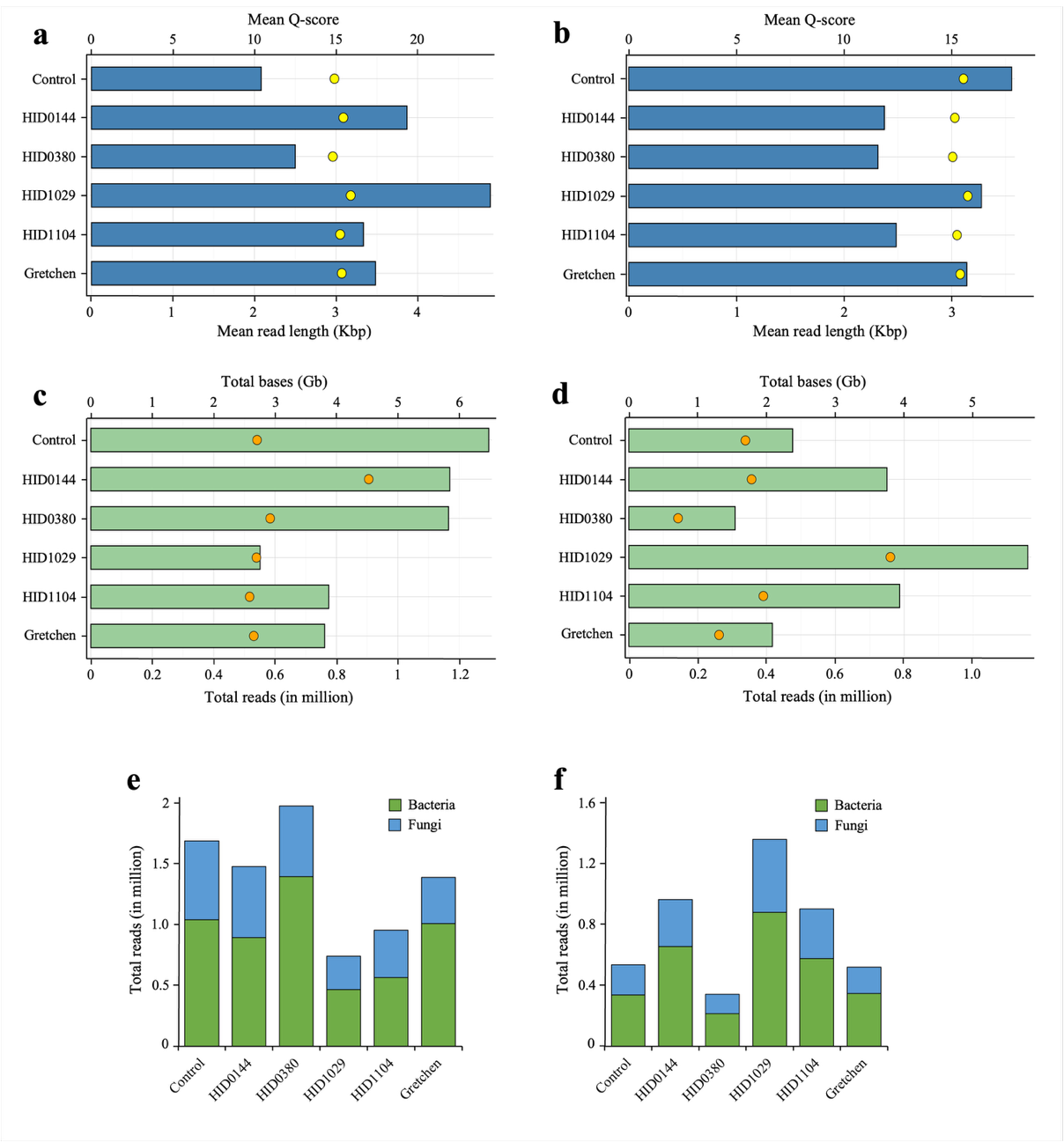


# **Figure S2. Oxford Nanopore whole-metagenome sequencing (WMS) statistics of rhizosphere microbiome samples from 2024 and 2025.**

**(a** and **b)** Mean read length (bars) and average Phred quality scores (yellow dots) for individual genotypes sequenced in 2024 **(a)** and 2025 **(b)**. **(c** and **d)** Total number of reads (bars) and cumulative sequencing yield (orange dots, in gigabases) obtained from each sample for 2024 **(c)** and 2025 **(d)**. **(e** and **f)** Relative proportions of classified bacterial (green) and fungal (blue) reads across samples from 2024 **(e)** and 2025 **(f)**, as determined by Kraken2 using the prebuilt bacterial and fungal genome databases. ONT long-read sequencing produced high-quality datasets suitable for metagenomic analysis, with bacterial reads comprising the majority of classified sequences in both years. Each genotype is represented by two biological replicates per year.


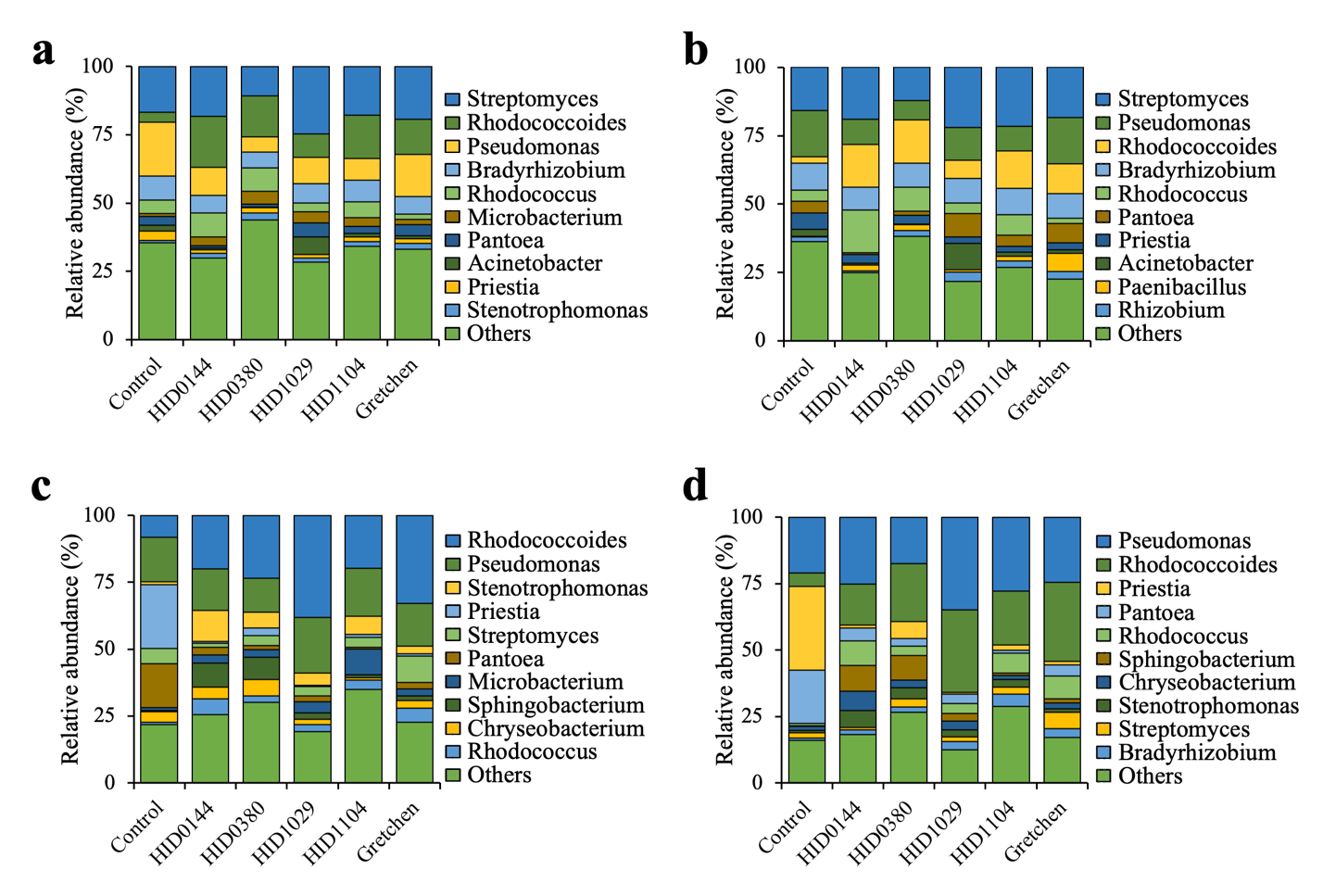


# **Figure S3.** **Effect of Kraken2 confidence thresholds on bacterial taxonomic profiles of barley rhizosphere metagenomes.**

Stacked bar plots show the relative abundance of the top 10 bacterial genera identified from Oxford Nanopore whole-metagenome sequencing using Kraken2 classification at increased confidence thresholds. **(a)** Bacterial genus composition for samples collected in 2024 classified with a confidence threshold of 0.5. **(b)** Bacterial genus composition for 2024 samples classified with a confidence threshold of 0.7. **(c)** Bacterial genus composition for samples collected in 2025 classified with a confidence threshold of 0.5. **(d)** Bacterial genus composition for 2025 samples classified with a confidence threshold of 0.7. Only classified reads were retained, and relative abundances were re-normalized to 100% after removal of unclassified reads. Across both years, increasing the confidence threshold reduced low-abundance taxa but preserved dominant genera and overall genotype-associated community patterns.


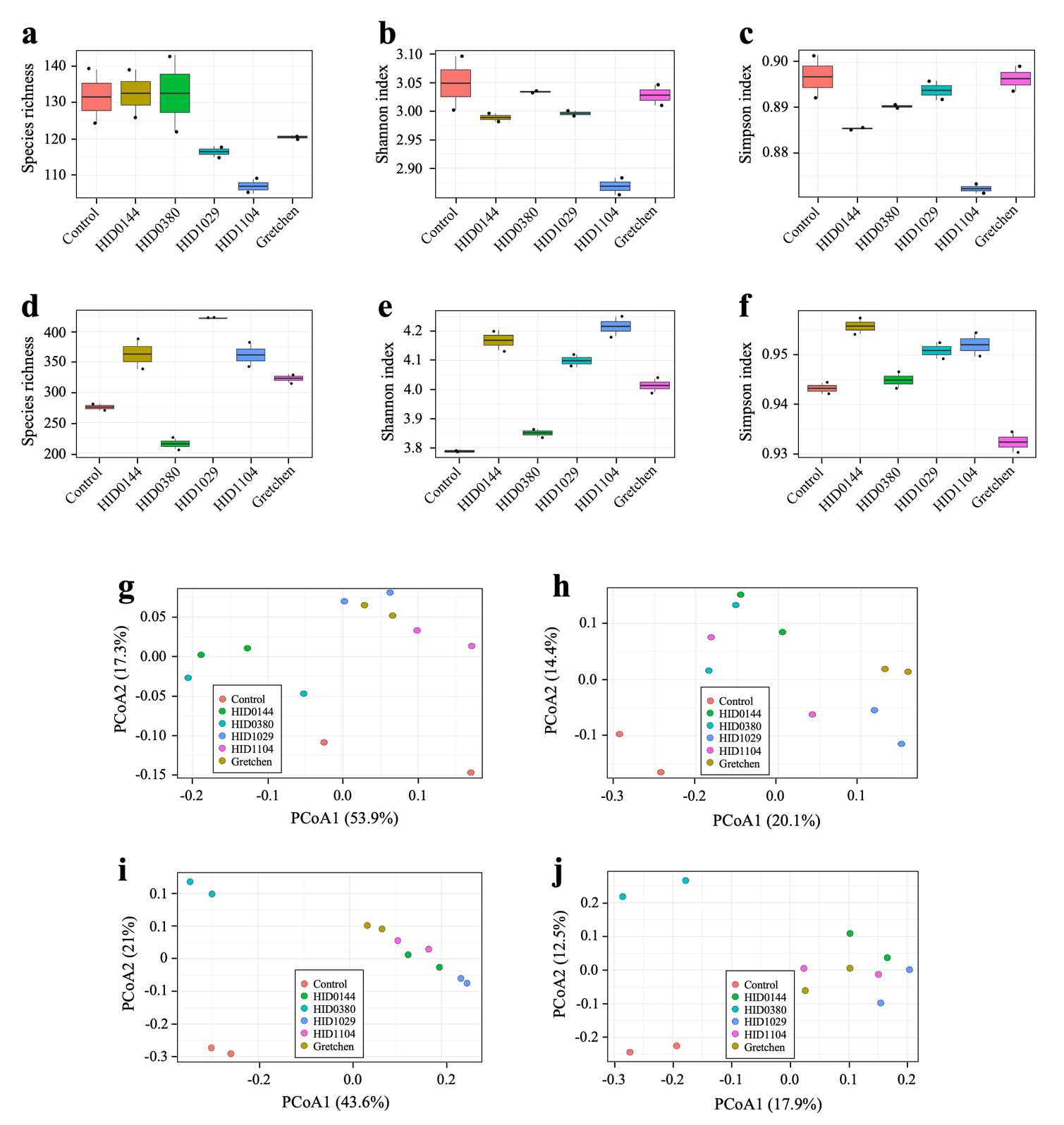


# **Figure S4. Alpha and beta diversity of WMS-derived bacterial communities in rhizosphere samples from 2024 and 2025.**

**(a - c)** Alpha diversity indices for 2024 samples, including species richness **(a)**, Shannon **(b)**, and Simpson **(c)** indices (Kruskal-Wallis, *p* > 0.05; Richness: χ² = 9.59, p = 0.0877). **(d - f)** Corresponding alpha diversity indices for 2025 samples (Kruskal-Wallis, *p* > 0.05; χ² = 10.50, p = 0.0623). **(a - f)** Across both years, no statistically significant differences were observed among barley genotypes, indicating comparable community richness and evenness. On average, 2025 samples exhibited higher genus richness (220 - 450 genera) than those from 2024 (100 - 150 genera), suggesting a more complex rhizosphere microbiome. **(g and h)** Principal Coordinate Analysis (PCoA) plots based on Bray-Curtis dissimilarity for 2024 **(g)** and 2025 **(h)**, reflecting differences in bacterial community composition driven by relative taxon abundance (R² = 0.79, F = 4.64, p = 0.001 in 2024; R² = 0.93, F = 16.91, p = 0.001 in 2025). **(i and j)** PCoA plots based on Jaccard distance for 2024 **(i)** and 2025 **(j)**, depicting compositional differences based on presence or absence of taxa (R² = 0.60, F = 1.84, p = 0.001 in 2024; R² = 0.56, F = 1.54, p = 0.001 in 2025). **(g - j)** PERMANOVA confirmed significant genotype-dependent community separation (Bray-Curtis p = 0.001; Jaccard p = 0.001).


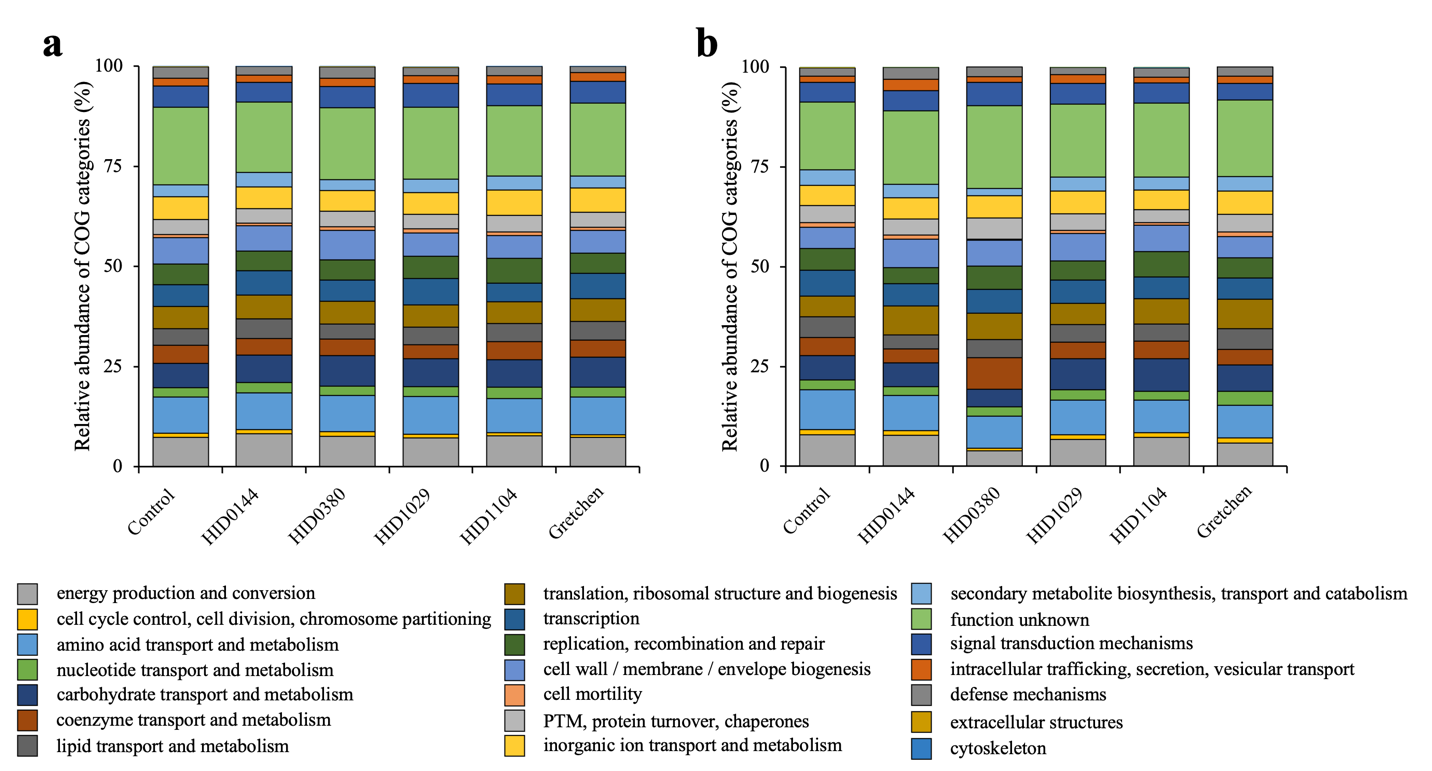


# **Figure S5. Functional categorization of predicted proteins from metagenomic assemblies across rhizosphere samples in 2024 and 2025.**

Predicted proteins obtained from Oxford Nanopore metagenomic assemblies were annotated using eggNOG-mapper to generate COG functional classifications for 2024 **(a)** and 2025 **(b)**. Stacked bar plots display the relative abundance of major COG categories across genotypes, revealing consistent functional distributions among samples and between years. The dominant categories included amino acid transport and metabolism, energy production and conversion, and cell wall/membrane/envelope biogenesis, indicating stable core metabolic functions within the rhizosphere microbiome. Approximately 20% of predicted proteins were annotated as hypothetical or of unknown function, reflecting the presence of uncharacterized microbial genes. Despite differences in the total number of predicted proteins among genotypes, the relative distribution of functional categories remained conserved, suggesting a functionally stable and metabolically balanced microbiome.


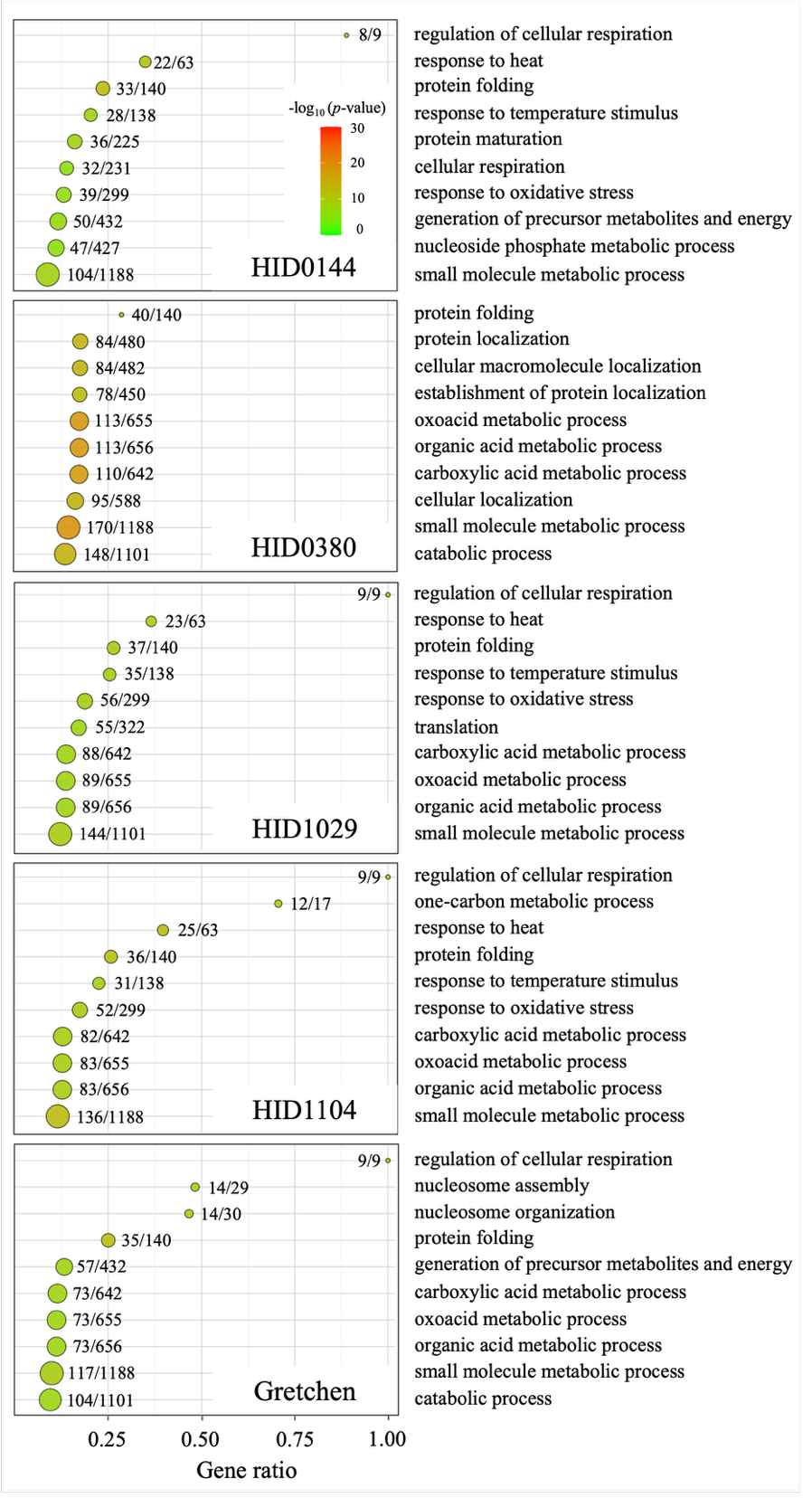


# **Figure S6. Gene Ontology (GO) enrichment analysis of differentially expressed genes (DEGs) across barley genotypes.**

Bubble plots showing the top ten enriched Gene Ontology biological process (GO:BP) terms for each genotype including wild accessions HID0144 and HID0380, landraces HID1029 and HID1104, and the modern cultivar Gretchen. The X-axis represents the gene ratio, defines as the number of DEGs associated with a GO term divided by the total number of genes annotated to that term. Bubble sizes indicate the number of DEGs mapped to each GO term, and color intensity corresponds to enrichment significance (-log₁₀ adjusted p-value). Only GO terms with adjusted *p-*value less than 0.05 are included. Across all genotypes, core biological processes including response to stress, small molecule metabolism, and regulation of cellular respiration were commonly enriched. Genotype-specific enrichments were also observed, such as nucleoside phosphate metabolism in HID0144, catabolic and energy-producing processes in HID0380 and Gretchen, and chromatin organization in Gretchen. The landraces HID1029 and HID1104 exhibited closely related enrichment profiles with variable enrichment magnitude.


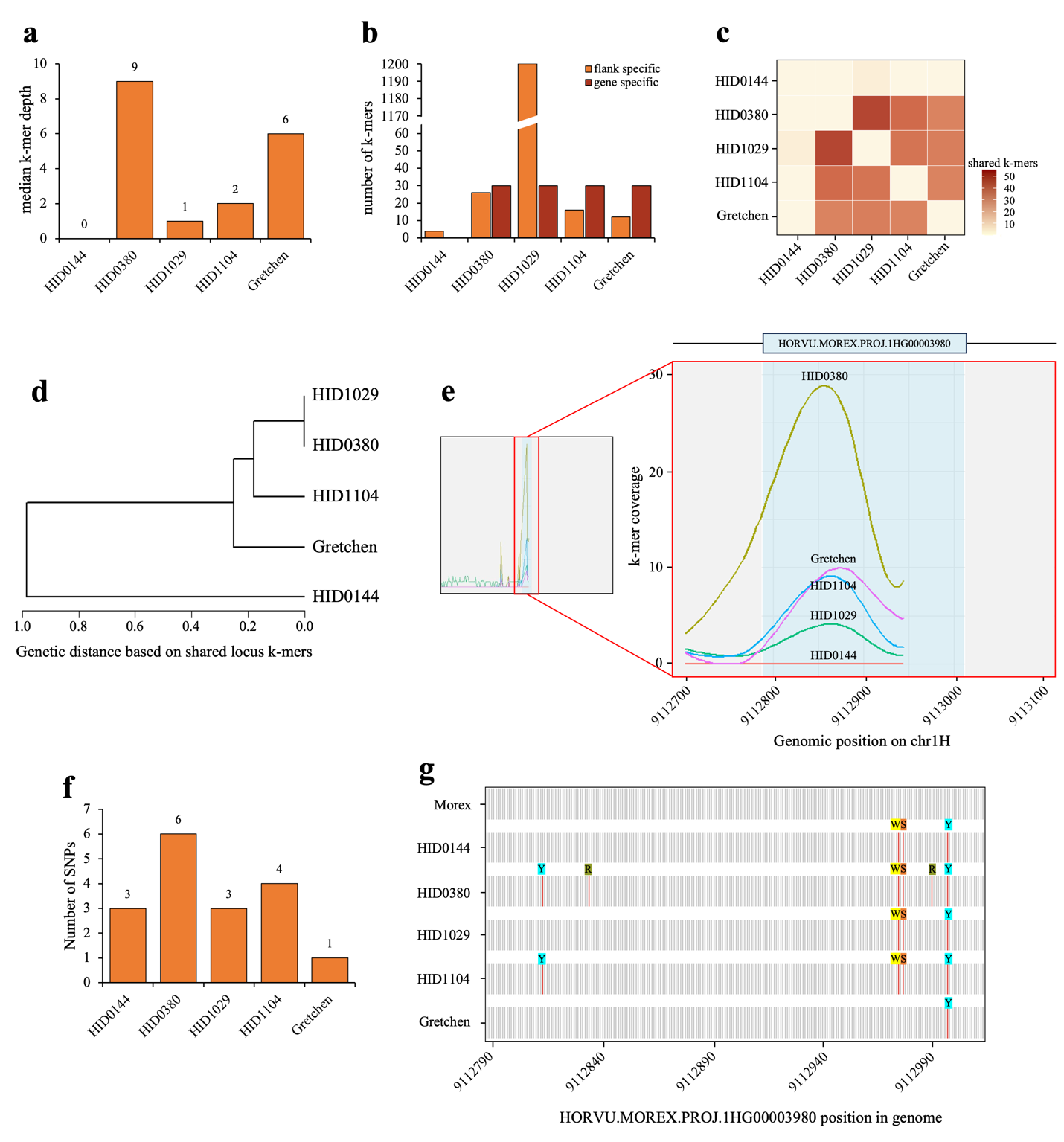


# **Figure S7. Structural and nucleotide-level variation at the HORVU.MOREX.PROJ.1HG00003980 locus across barley genotypes.**

**(a)** Median *k*-mer depth across the gene shows strong dosage differences, with HID0380 displaying the highest depth and HID0144 showing no detectable Morex-like sequence, indicating absence or strong divergence of the gene. **(b)** Gene-specific and locus-wide *k*-mer counts reveal that all genotypes except HID0144 retain the full set of gene-specific *k*-mers (30), whereas flank-derived *k*-mers vary widely, with HID1029 showing the highest locus similarity to Morex. **(c)** Heatmap of pairwise shared *k*-mers illustrates varying degrees of locus similarity, with HID0380 and HID1029 sharing the most *k*-mers. **(d)** Hierarchical clustering of locus-wide *k*-mer similarity groups HID0380 and HID1029 as the closest pair, while HID0144 forms a distinct outgroup. **(e)** Spatial *k*-mer coverage profiles highlight a strong peak in HID0380 and progressively lower coverage in HID1104, HID1029, and Gretchen, consistent with differences in Morex-like sequence dosage, illustrating localized structural variation of the gene. **(f)** Bar plot showing the number of SNPs detected within the 224 bp coding region of HORVU.MOREX.PROJ.1HG00003980. HID0380 carries the highest number of nucleotide substitutions (6), followed by HID1104 with 4 SNPs, whereas HID0144 and HID1029 each harbor 3 SNPs, and Gretchen shows only a single SNP. **(g)** Per-base SNP visualization for each genotype aligned to Morex. Grey marks indicate positions identical to the reference, while red marks indicate SNPs. Above each SNP, an **IUPAC ambiguity code** denotes the alternative nucleotide(s) present in the genotype. Codes represent: **R (A/G), S (C/G), W (A/T), Y (C/T).**
